# Analysis of Genomic DNA from Medieval Plague Victims Suggests Long-Term Effect of *Yersinia pestis* on Human Immunity Genes

**DOI:** 10.1093/molbev/msab147

**Published:** 2021-05-18

**Authors:** Alexander Immel, Felix M Key, András Szolek, Rodrigo Barquera, Madeline K Robinson, Genelle F Harrison, William H Palmer, Maria A Spyrou, Julian Susat, Ben Krause-Kyora, Kirsten I Bos, Stephen Forrest, Diana I Hernández-Zaragoza, Jürgen Sauter, Ute Solloch, Alexander H Schmidt, Verena J Schuenemann, Ella Reiter, Madita S Kairies, Rainer Weiß, Susanne Arnold, Joachim Wahl, Jill A Hollenbach, Oliver Kohlbacher, Alexander Herbig, Paul J Norman, Johannes Krause

**Affiliations:** 1 Max Planck Institute for the Science of Human History, Jena, Germany; 2 Institute of Clinical Molecular Biology, Kiel University, Kiel, Germany; 3 Institute of Archaeological Sciences, University of Tübingen, Tübingen, Germany; 4 Max Planck Institute for Infection Biology, Berlin, Germany; 5 Applied Bioinformatics, Department for Computer Science, University of Tübingen, Tübingen, Germany; 6 Division of Biomedical Informatics and Personalized Medicine, and Department of Immunology & Microbiology, University of Colorado, Boulder, CO, USA; 7 Immunogenetics Unit, Técnicas Genéticas Aplicadas a la Clínica (TGAC), Mexico City, Mexico; 8 DKMS, Tübingen, Germany; 9 Institute of Evolutionary Medicine, University of Zurich, Zurich, Switzerland; 10 Institute for Archaeological Sciences, WG Palaeoanthropology, University of Tübingen, Tübingen, Germany; 11 State Office for Cultural Heritage Management, Stuttgart Regional Council, Esslingen, Germany; 12 UCSF Weill Institute for Neurosciences, Department of Neurology, University of California, San Francisco, CA, USA; 13 Institute for Bioinformatics and Medical Informatics, University of Tübingen, Tübingen, Germany; 14 Quantitative Biology Center, University of Tübingen, Tübingen, Germany; 15 Translational Bioinformatics, University Hospital Tübingen, Tübingen, Germany; 16 Biomolecular Interactions, Max Planck Institute for Developmental Biology, Tübingen, Germany; 17 Max Planck Institute for Evolutionary Anthropology, Leipzig, Germany

**Keywords:** ancient DNA, aDNA, HLA, plague, Yersinia pestis, human immunity, natural selection

## Abstract

Pathogens and associated outbreaks of infectious disease exert selective pressure on human populations, and any changes in allele frequencies that result may be especially evident for genes involved in immunity. In this regard, the 1346-1353 *Yersinia pestis*-caused Black Death pandemic, with continued plague outbreaks spanning several hundred years, is one of the most devastating recorded in human history. To investigate the potential impact of *Y. pestis* on human immunity genes, we extracted DNA from 36 plague victims buried in a mass grave in Ellwangen, Germany in the 16th century. We targeted 488 immune-related genes, including *HLA*, using a novel in-solution hybridization capture approach. In comparison with 50 modern native inhabitants of Ellwangen, we find differences in allele frequencies for variants of the innate immunity proteins Ficolin-2 and NLRP14 at sites involved in determining specificity. We also observed that HLA-DRB1*13 is more than twice as frequent in the modern population, whereas *HLA-B* alleles encoding an isoleucine at position 80 (I-80+), HLA C*06:02 and* HLA-DPB1* alleles encoding histidine at position 9 are half as frequent in the modern population. Simulations show that natural selection has likely driven these allele frequency changes. Thus, our data suggest that allele frequencies of *HLA* genes involved in innate and adaptive immunity responsible for extracellular and intracellular responses to pathogenic bacteria, such as *Y. pestis*, could have been affected by the historical epidemics that occurred in Europe.

## Introduction

Throughout evolution, humans have likely experienced multiple major episodes of infectious disease. Of exceptional virulence and lethality, *Yersinia pestis* has been responsible for at least three major plague pandemics during the last few millennia. Studies of ancient DNA have confirmed *Y. pestis* caused widespread infections in Europe from the Late Neolithic period, nearly 5,000 years ago, until the AD 18th century (Bos et al. 2011, 2016; Wagner et al. 2014; Rasmussen et al. 2015; Andrades Valtuena et al. 2016; Feldman et al. 2016; Namouchi et al. 2018; Spyrou et al. 2018; Keller et al. 2019; Rascovan et al. 2019). Historical records show the first pandemic began with the Justinianic Plague in the AD 6th century and lasted until the 8^th^ century, the second began with the 1346–1353 Black Death and continued with thousands of local plague outbreaks until the 18th century (Biraben 1976; Büntgen et al. 2012), and the third pandemic started in China in the AD 19th century and spread the pathogen worldwide lasting up until the mid-20th century (Politzer 1954; Morelli et al. 2010). Of the three recorded pandemics, the Black Death claimed up to half of the European population during its 5-year period (Benedictow 2004). Although *Y. pestis* is now absent from most of Europe, it still causes sporadic infections among humans in the Americas, Africa, and Asia, usually transmitted by fleas from rodent populations that serve as plague reservoirs (Drummond et al. 2014). Although the lethality of plague is very high without treatment, it remains likely that specific individuals are protected from, or more susceptible to, severe disease through polymorphism in the determinants of natural immunity. In this case, any changes in allele frequencies that occurred during a given epidemic crisis could be evident as genetic adaptation and detectable in modern-day individuals.

There are multiple examples of natural selection affecting human immunity-related genes that can be attributed to challenge by pathogens. These examples include specific pathogens causing malaria, cholera, or Lassa fever, or to wider differences in pathogen exposure between geographically discrete populations (Kwiatkowski 2005; Voight et al. 2006; Wang et al. 2006; Sabeti et al. 2007; Karlsson et al. 2013; Key et al. 2014; McManus et al. 2017; Harrison et al. 2019). The toll-like receptors (TLRs) are innate immune proteins that detect the presence of specific pathogens to initiate an immune response. Signatures of purifying selection have been identified within specific *TLR* genes that correlate with distinct pattern specificities of the encoded allotypes (Barreiro et al. 2009). In another example, recent signatures of positive selection in the *IFITM3* gene accompany differential abilities of the alternative variants to control pandemic H1N1 influenza A virus infection (Albright et al. 2008; Everitt et al. 2012). A final example of human genetic adaptation to pathogens is the 32-bp deletion in the chemokine receptor *CCR5* (*CCR5-Δ32*), which prevents HIV from entering and infecting human T cells (Dean et al. 1996). Although once postulated as a plague resistance allele (Stephens et al. 1998), there is little evidence for positive selection acting on *CCR5-Δ32* (Sabeti et al. 2006). By contrast, the major histocompatibility complex (*MHC*), which encodes multiple immunity-related genes including the human leukocyte antigen (HLA) molecules, does show evidence for recent positive and balancing selection and has established roles initiating and directing the immune response to infection (Prugnolle et al. 2005; Parham and Moffett 2013; Trowsdale and Knight 2013; Klebanov 2018).

Here, we extracted genomic DNA from 36 individuals who apparently died from plague (*Y. pestis*) in Ellwangen in Southern Germany during the 16th century. We also extracted DNA from 50 modern-day Ellwangen inhabitants. We then compared their frequency spectra for a large panel of immunity-related genes. We observed evidence for pathogen-induced changes in allele distributions for two innate pattern-recognition receptors and four HLA molecules. We propose that these frequency changes could have resulted from *Y. pestis* plague exposure during the 16th century.

## Results

### Archaeological and Anthropological Findings

**Fig. 1.**
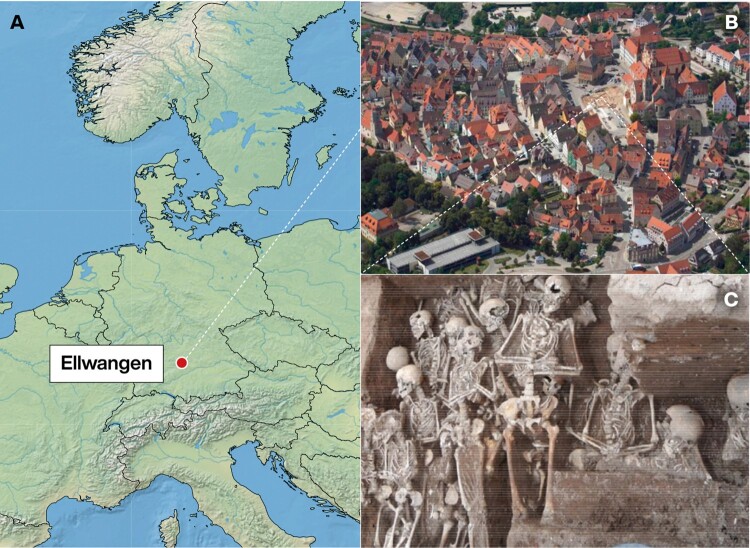
Mass burials discovered at Ellwangen. (*A*) Location of Ellwangen in Germany. (*B*) Location of the marketplace, where the mass burials were discovered during an excavation in 2013–2015. (*C*) Mass grave 549 showing several individuals being buried together.

Ellwangen is a small town of 27,000 inhabitants situated in South Germany near the border of Baden-Wuerttemberg and Bavaria. Ellwangen was founded in the AD 7th century, with only a few hundred inhabitants until modern times. The town was affected by multiple plague outbreaks during the 16th and 17th centuries (Ellwangen 2007). From 2013 to 2015 an excavation took place in Ellwangen during the restoration of the town’s market square (fig. 1). Three mass graves were discovered with a total of 101 inhumated remains (supplementary fig. 1*A*, Supplementary Material online). Consistent with 16th century bubonic plague predominantly affecting children (Bowsky 1971; Clouse 2002; Cohn 2003) only 23 of the individuals had reached adult age (supplementary note 1, Supplementary Material online). The individuals were buried close to each other and there was little sediment between the distinct layers. The proximity, as well as radiocarbon dating, suggests that all three mass burials were created during the same epidemic crisis event during the 16th century (supplementary fig. 1*B*, Supplementary Material online). Genomic DNA from *Y. pestis* was identified previously from 13 of the individuals, and the complete genome of a strain consistent with the era was reconstructed from one of them (Spyrou et al. 2016, 2019). We also performed shotgun sequencing directly on DNA libraries prepared from tooth samples of 30 distinct individuals, and pathogen screening using the metagenomic alignment tool MALT (Vågene et al. 2018) identified reads matching to *Y. pestis* in 25 of them, with aDNA characteristic terminal substitutions in samples with sufficient coverage (supplementary table 1, Supplementary Material online), confirming that the reads are of ancient DNA origin. With exception of one sequence read of Hepatitis B Virus in one of the petrous bone samples (ELW012), no evidence of other pathogens was detected. Additional archaeological and anthropological findings suggest little physical trauma, albeit poor health condition prior to death, which can likely be considered as normal health and nutrition status for people living in that time period (supplementary note 1, Supplementary Material online). Taken together, these findings strongly suggest that these individuals were victims of a single *Y. pestis* plague outbreak that occurred during the 16th century.

### The 16th Century Ellwangen Plague Victims Display Genetic Similarity with Modern Inhabitants

**Fig. 2.**
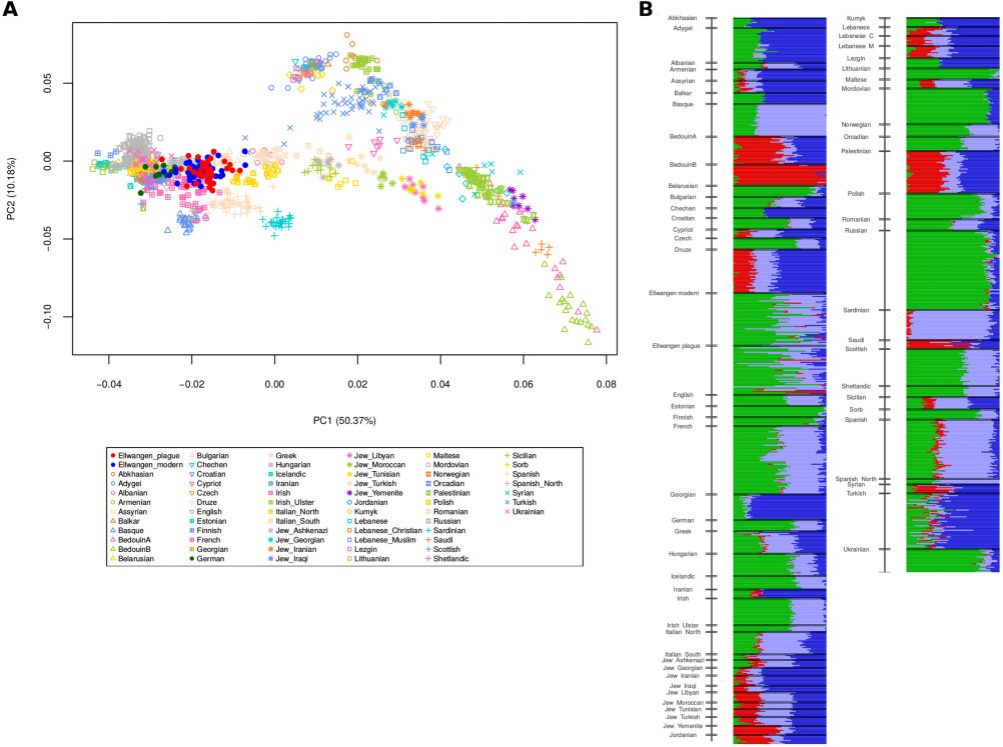
The 16th century plague victims and modern inhabitants of Ellwangen form a continuous population. (*A*) PCA showing the 16th century (red) and modern (blue) Ellwangen populations in the context of 65 modern-day populations from West-Eurasia based on 1,233,013 genome-wide SNPs (Lazaridis et al. 2014; Haak et al. 2015; Fu et al. 2016). (*B*) Admixture modeling based on four ancestral components (*K* = 4) of the same 65 modern West Eurasian populations including 16th century (Ellwangen plague) and modern Ellwangen (Ellwangen modern) populations. The *K* = 4 model was chosen due to the lowest cross-validation error.

From the 16th century mass grave site in Ellwangen, we successfully extracted DNA from 40 petrous bones (supplementary fig. 1*C*, Supplementary Material online) and four teeth. DNA of sufficient quality and quantity for genome-wide sequence analysis was obtained from all samples. An average of 1.76 million unique, human genome reads per individual was generated by shotgun sequencing (supplementary data 1A, Supplementary Material online). Kinship analysis revealed three pairs of individuals to be first-degree relatives (supplementary data 1B and fig. 2, Supplementary Material online). In order to obtain the most accurate frequency distributions, one in each pair of the directly related individuals was removed from the allele frequency calculations. In these cases, the individual having the lowest yield of sequence reads was excluded (Materials and Methods). In addition, one individual who was second-degree related to two of the other individuals was also excluded (ELW030). We also obtained genomic DNA samples from 51 contemporary inhabitants of Ellwangen and shotgun-sequenced them with an average of 2.74 million unique human genome reads per individual (supplementary data 1A, Supplementary Material online). Here, we identified a single pair of first-degree relatives and removed one individual. In order to test whether the two cohorts derive from a single continuous population, we tested for population genetic similarity using principle component analysis (PCA; Patterson et al. 2006) and ADMIXTURE analysis (Alexander et al. 2009). Showing that the 16th century and modern groups indeed are genetically very similar, we found that the 16th century Ellwangen plague victims form a tight cluster in PCA space, which overlaps with the modern inhabitants (fig. 2*A*). This finding is bolstered by the highly similar genetic ancestry composition of the two groups as illustrated by their population admixture proportions (fig. 2*B*). This latter finding is important because recent demographic changes could alter allele frequencies of the modern compared with the 16th century group (Hellenthal et al. 2014).

### Two Immunity-Related Genes Harbor Strongly Differentiated single nucleotide polymorphisms

In order to compare the allele spectra of immunity-related genes in the 16th century *Y. pestis* plague victims with modern-day inhabitants of Ellwangen, we developed an in-solution hybridization capture approach to enrich for 488 human genes implicated in immunity (supplementary table 2, Supplementary Material online). This approach allowed us to specifically target the genes of interest while reducing the amount of sequencing required, leading to an average of 308 times more reads on target compared with undirected genome wide sequencing (supplementary fig. 3, Supplementary Material online). We applied this “immunity capture” method to all 16th century and modern DNA samples. The targeted genes were covered with a mean read depth of 55.8 (supplementary data 2, Supplementary Material online). We investigated the allele spectra of the 488 immunity-related genes by leveraging a branching statistic, *Differentiation with Ancestral* (*DAnc*) (Key et al. 2016). *DAnc* is calculated per site and uses derived allele frequency (DAF) estimates across three populations; 16th century and modern Ellwangen, and a non-European outgroup (we used Han Chinese from Beijing; Abecasis et al. 2012). *DAnc* scores can range from −1 to +1, and those in the respective far tails of the distribution identify candidates for simulation studies that could indicate positive selection has occurred. We established the expected distribution of *DAnc* scores (supplementary data 3, Supplementary Material online) under neutrality through simulations using a human demographic model (Gravel et al. 2011).

In our analysis, the distribution of *DAnc* scores closely matches between the simulated and test data (supplementary fig. 4 and table 3, Supplementary Material online). In the far tail of the distribution (>99.9%), we observed three single nucleotide polymorphisms (SNPs), two in the Ficolin-2 (*FCN2*) gene and one in the NOD-like receptor purine domain containing 14 (*NLRP14*) gene (table 1) also corresponding to the greatest *F_ST_* values among the 488 genes for the same three SNPs (supplementary data 4, Supplementary Material online). *F_ST_* is an established measure for population differentiation and corrects for expected heterozygosity and sampling error (Weir and Cockerham 1984). However, due to the ascertainment of SNPs, which are not representative of the whole genome, the far tail of the observed DAnc or *F_ST_* distribution is no evidence alone for positive selection. Alternatively, we compared the fraction of SNPs observed in the far tail of the simulated and test distribution, which suggests no enrichment of SNPs in our test data and thus no evidence for positive selection using the data at hand (supplementary table 3, Supplementary Material online). Further analyses using a larger sample size and whole-genome data are necessary in order to understand the role of positive selection due to historic epidemics.

**Table 1.** Genes Identified in the 0.01% Tail of Distribution Following *DAnc* Analysis.

Chromosome			Allele			Ellwangen									
No	Position	SNP ID	Ref	Alt	Der	Anc	Mod	CEU	FIN	GBR	DAnc	*F_ST_* empirical*P* value	Variant	Gene	Function
9	137772664	rs17514136	A	G	G	0.17	0.37	0.28	0.24	0.21	0.20	0.99	5_prime_UTR	FCN2	Activation of thelectin complementpathway
9	137779026	rs17549193	C	T	T	0.19	0.39	0.29	0.25	0.23	0.20	0.99	Missense	FCN2	
11	7079038	rs10839708	G	A	A	0.69	0.51	0.60	0.65	0.63	0.18	0.97	Missense	NLRP14	Activation ofproinflammatorycaspases

Note.—Ref, reference; Alt, alternative; Der, derived; Anc, DAF in Ellwangen 16th century; Mod, DAF in Ellwangen modern; CEU, DAF in Central Europeans from Utah; FIN, DAF in Finnish; GBR, DAF in Great Britains (obtained from the 1000 Genomes Project Phase 3 data). Shown are alleles that have significantly (*P* < 0.05) changed in frequency in the modern individuals. *F_ST_* empirical *P* value refers to the empirical distribution of *F_ST_* calculated between the 16th century and the modern Ellwangen population.

The identified SNPs of FCN2 are a 5ʹ UTR promoter variant (rs17514136 [-4 A to G]) and one coding change variant (rs17549193 [717 C to T; 236 Thr to Met]). The UTR and coding change variants occur in complete linkage disequilibrium (LD; Δ' = 1.0, *R*^2^ = 0.9), and appear to represent a single haplotype that has risen in frequency in the modern population. Interestingly, FCN2 binds to specific molecules on the surface of bacteria, triggering the complement pathway to neutralize the pathogen (Hoang et al. 2011; Luo et al. 2013). The promoter variant is associated with increased serum concentration of FCN2 (Cedzynski et al. 2007), whereas polymorphism at residue 236 (rs17549193) affects binding to the target bacteria (Hummelshoj et al. 2005). Similarly, NLRP14 belongs to inflammasome complex proteins, which are intracellular pattern recognition receptors that trigger local and systemic responses to microbial invasion (Martinon et al. 2002). Inflammasomes are implicated in the immune response to *Yersinia* infection, amongst other pathogens (Vladimer et al. 2013; Philip et al. 2016). The *NLRP14* SNP is a coding change variant (rs10839708 [2745 G to A: 808 Glu-Lys]) that occurs in the leucine-rich repeat (LRR) domain, which in related molecules controls the ligand specificity (Inohara et al. 2005). Thus, in summary, we show immune-related genes have no significant frequency changes between 16th century *Y. pestis* victims and modern Ellwangen inhabitants.

### No Evidence for Role of CCR5-Δ32 in Protection from Y. pestis Infection

We investigated the Δ32 deletion in the *CCR5* locus (chr3:46414947-46414978), which was included in our target regions because this mutation has previously been suggested as protective from the plague. We found that *CCR5-Δ32* has a frequency of 16.6% in the 16th century compared with 10.8% in the modern individuals (*P* = 0.27) and 11.2% in Germany (supplementary data 5A, B and 6A, Supplementary Material online; table 2). Consistent with epidemiological modeling and lack of evidence that CCR5 can serve as a *Y. pestis* receptor (Galvani and Slatkin 2003), this finding suggests that the *CCR5-Δ32* mutation provided no protection from *Y. pestis*. Similarly, we also investigated SNPs *rs4986790*, *rs4986791* within the gene *TLR4* previously suggested to be associated with resistance to *Y. pestis* (Laayouni et al. 2014; Al Nabhani et al. 2017). However, we did not find any significant differences in their respective frequencies (supplementary table 4, Supplementary Material online).

**Table 2.** Genotype and Allele Frequencies of *CCR5*-Wild type (wt), and *CCR5-Δ32* (Δ32), among the Plague Victims and Modern Individuals from Ellwangen.

	Genotype frequency (%)				Allele frequency (%)	
	wt/wt	wt/Δ32	Δ32/Δ32	Wt		Δ32
**Ellwangen plague**	71.4	23.8	4.8	83.4		16.6
**Ellwangen modern**	78.4	21.6	0	89.2		10.8
**Germany**	79.2	19.4	1.4	88.8		11.2

Note.—The individual genotypes are given in supplementary data 6A, Supplementary Material online. The frequencies for Germany were obtained from a study of German bone marrow donor registry volunteers (Solloch et al. 2017) for comparison.

### Natural Selection Has Increased HLA-DRB*13 and Reduced HLA-B*51 and -C*06 Frequencies in Modern Individuals

With more than 28,000 distinct alleles described (Robinson et al. 2015), HLA molecules are encoded by the most polymorphic gene complex in humans. When human populations are exposed to novel diseases through contact with populations or environments they had not encountered previously, changes in *HLA* allele frequencies can occur rapidly (Lindo et al. 2016; Patin et al. 2017). Consequently, the signatures of balancing selection in the genomic region that contains *HLA* are consistently the strongest in the genome (Sabeti et al. 2006; Quintana-Murci 2019), and specifically correspond to amino acid residues that bind peptide fragments derived from pathogens (Bjorkman and Parham 1990). Significant shifts in *HLA* allele frequencies can thus reveal evidence of natural selection for specific pathogen resistance. We were able to identify *HLA class I (-A, -B, -C)* and *HLA class II (-DPA1*, *-DPB1*, *-DQA1*, *-DQB1*, and *-DRB1)* genotypes from all of the 16th century and modern inhabitants of Ellwangen. We observed a total of 86 distinct *HLA class I* alleles, 66 distinct *HLA class II* alleles, and 168 distinct *HLA* haplotypes (supplementary data 6B, Supplementary Material online). The most frequent haplotype (HLA-A*01:01∼B*08:01∼C*07:01∼DRB1*03:01) is the same in both groups and is also the most common and widespread across Europe today (Darke et al. 1998; Dunne et al. 2008; Johansson et al. 2008; Nowak et al. 2008; Pingel et al. 2013). Thus, the diversity and composition of *HLA* haplotypes appear as expected for Northern European populations (Alfirevic et al. 2012), and we did not observe any significant differences in their frequencies between the 16th century and modern individuals.

By contrast to the haplotype distributions, on examining the individual *HLA class I* genes, we observed that the B*51:01 allele of *HLA-B* decreased from 15.3% in the 16th century Ellwangen plague victims to only 6.0% (*P* = 0.04 [*P*-corrected = NS]; DANc = −0.093) in the modern Ellwangen population (table 3, supplementary data 4 and 5A, Supplementary Material online). Similarly, the C*06:02 allele of *HLA-C* decreased from 13.9% to 5% (*P* = 0.04 [*P*-corrected = NS]; DANc = 0.053). HLA-B*51:01 and -C*06:02 are not in LD in either population (supplementary data 6B, Supplementary Material online), and so these two observations are independent. In addition, although there were no significant frequency differences observed for any *HLA class II* alleles as determined at two-field resolution, we observed that all allotypes present representing the DR13 serological group (Holdsworth et al. 2009) were at substantially lower frequency in the 16th century than modern Ellwangen population. Accordingly, by considering them together, there was an increase in *DR13* frequency from 5.6% in the 16th century to 17.0% in the modern individuals (*P* = 0.026, table 3, supplementary data 5A, Supplementary Material online). Repeating this analysis for all the major *DRB1* lineages present (Holdsworth et al. 2009), showed DRB1*13 as the only allotype differing in frequency between the two groups (table 3). We used Wilson Score Interval estimation of the 95% binomial confidence interval (CI). The 95% CI of HLA-B*51:01 was 0.09–0.25 (observed = 0.06), the 95% CI of HLA-C*06:02 was 0.08–0.24 (observed = 0.05), and DRB1*13 was 0.02–0.13 (observed = 0.16). Thus, for each of the three HLA allotypes showing distinctions between modern and 16th century inhabitants of Ellwangen, the observed modern allele frequencies are outside the 95% binomial confidence intervals surrounding sampling of the 16th century allele frequencies. We further validated these findings by comparing the *HLA* allele frequencies observed in the Ellwangen individuals with a large panel (*N* = 8,862) of unrelated bone marrow donor registry volunteers gathered from all of Germany (supplementary data 5B, Supplementary Material online). Whereas there were no significant allele frequency differences when comparing modern inhabitants of Ellwangen with modern Germany as a whole, we observed significantly lower frequencies of B*51:01 in modern Germany (5.5%) than the Ellwangen plague victims (15.3%), when applying a pairwise proportion test (*P* = 0.005; DANc = −0.098). We also observed differences in HLA-C*06 and DRB1*13 between the plague victims and modern Germany, but these were not statistically significant (supplementary data 5B, Supplementary Material online).

**Table 3.** *HLA-B*, *-C*, and *-DRB1* Allele Frequencies in 16th Century Plague Victims and Modern Inhabitants of Ellwangen.

Locus	Frequency (%) Plague	Frequency (%) Modern	*P* value
B*07:02	13.89	14	0.984
B*08:01	9.72	13	0.508
B*13:02	1.39	3	0.490
B*14:02	1.39	4	0.315
B*15:01	5.56	10	0.294
B*18:01	5.56	3	0.402
B*27:05	4.17	3	0.680
B*35:01	4.17	6	0.595
B*35:03	4.17	2	0.403
B*38:01	2.78	1	0.379
B*40:01	4.17	2	0.403
B*44:02	4.17	6	0.595
B*49:01	1.39	1	0.814
B*50:01	4.17	1	0.174
B*51:01	**15.28**	**6**	**0.044**
B*52:01	1.39	1	0.814
B*57:01	4.17	2	0.403
C*01:02	4.17	2	0.403
C*02:02	4.17	3	0.680
C*03:03	4.17	11	0.106
C*03:04	6.94	4	0.393
C*04:01	11.11	15	0.460
C*05:01	2.78	5	0.467
C*06:02	**13.89**	**5**	**0.041**
C*07:01	13.89	17	0.580
C*07:02	15.28	12	0.533
C*07:04	1.39	1	0.814
C*08:02	1.39	4	0.315
C*12:02	1.39	1	0.814
C*12:03	5.56	5	0.871
C*15:02	5.56	4	0.632
DRB1*01:01	9.72	7	0.520
DRB1*01:02	1.39	2	0.763
DRB1*03:01	11.11	11	0.982
DRB1*04:01	9.72	3	0.063
DRB1*04:07	1.39	1	0.814
DRB1*04:08	1.39	2	0.763
DRB1*07:01	11.11	12	0.857
DRB1*09:01	1.39	2	0.763
DRB1*11:01	9.72	6	0.363
DRB1*11:03	2.78	2	0.738
DRB1*11:04	1.39	4	0.315
DRB1*12:01	1.39	1	0.814
DRB1*13:01	2.78	10	0.067
DRB1*13:02	2.78	6	0.323
DRB1*15:01	18.06	15	0.592
DRB1*15:02	1.39	1	0.814
DRB1*16:01	1.39	3	0.490
DRB1*01	11.11	9	0.625
DRB1*03	11.11	11	0.956
DRB1*04	15.28	10	0.281
DRB1*07	11.11	12	0.883
DRB1*11	13.89	13	0.838
DRB1*13	**5.56**	**17**	**0.026**
DRB1*15	19.44	16	0.529

Note.—Frequency differences with *P* < 0.05 are highlighted in bold. No significance could be obtained after multiple testing correction (for details see supplementary data 5A, Supplementary Material online).

**Table 4.** (A) Allele Frequencies for (Top) Presence of *KIR3DL1* Gene, (bottom) *KIR3DL1* Alleles (^ǂ^) Indicates Allele Not Expressed at the Cell Surface (Guethlein et al. 2015) and (B) Genotype Frequencies for *KIR3DL1 and I80^+^HLA-B* in 16th Century (plague) and Modern Inhabitants of Ellwangen.

(A)		Frequency plague	Frequency modern	
**Gene**	*KIR3DL1*	0.81	0.74	
	*00101	0.25	0.18	
	*002	0.17	0.13	
	*00401 ^‡^	0.11	0.08	
**Alleles**	*00501	0.13	0.18	
	*007	0.03	0.02	
	*008	0.07	0.04	
	*01502	0.06	0.11	

**(B)**		**Number observed**	*P* **=**	

**Genotype**	**Plague (*N* = 36)**	**Modern (*N* = 50)**	**(chi)**	**(lme)**
*I-80^+^ HLA-B*	20 (55.6%)	16 (32%)	0.029	0.07
*KIR3DL1*	35 (97.2%)	44 (88%)	0.099	0.29
*KIR3DL1 + I-80+ HLA-B*	19 (52.8%)	13 (26%)	0.011	0.01

Note.—Shown are *P* values from (chi) chi-square, and (lme) logistic mixed-effects model.

**Fig. 3.**
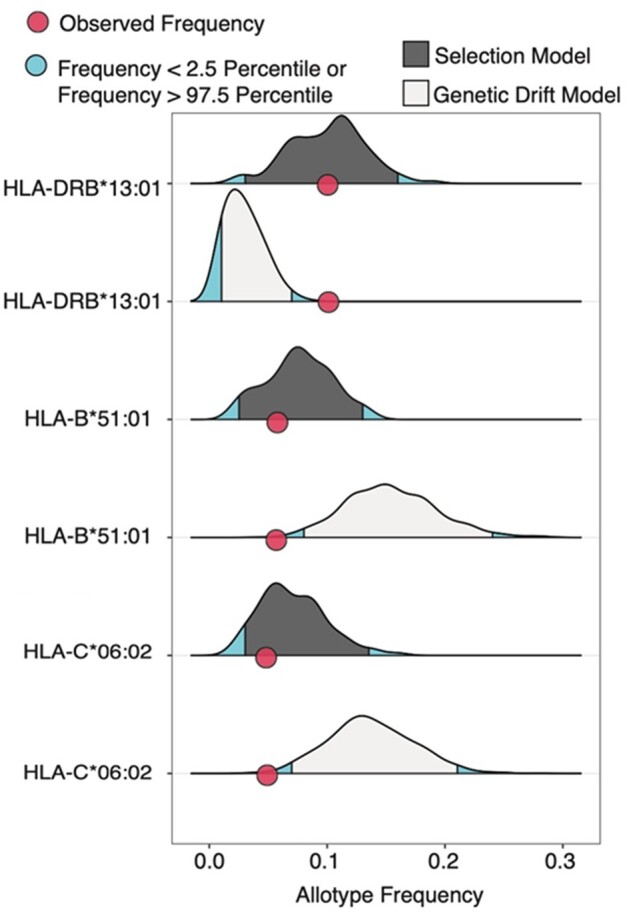
Natural selection drives HLA allele frequency changes. Density plots showing the distributions of allele frequencies from SLIM3 model simulations with (dark grey) or without (light grey) natural selection. The starting frequency for simulations was the observed frequency in the 16th century population. Selection coefficients for the models with natural selection were −0.1 for HLA-B*51:01 and HLA-C*06:02 and 0.2 for DRB1*13:01 (supplementary fig. 5, Supplementary Material online). The 2.5% extremes are shown in blue illustrating where the *P* value cutoff of 0.05 would occur. Red points represent the frequency in the modern-day population.

To distinguish if the changes in frequencies of B*51:01, C*06:02, and DRB1*13:01 were more likely to be due either to natural selection or genetic drift, we performed forward time simulations by starting from the observed polymorphisms in the 16th century Ellwangen and modeling neutrality for the last 500 years. This way it was possible to start from reasonable levels of genetic variation without the necessity to determine the impact of ancient selection on HLA and episodic turnover of HLA alleles. Moreover, this way each allotype could be tested individually. Again, we observed an overall concordance between median frequencies of the simulated neutral alleles and the modern Ellwangen allele frequencies, as is expected under genetic drift. By contrast, the allele frequencies of B*51:01, C*06:02, and DRB1*13:01 observed for modern inhabitants of Ellwangen were in the extreme tails of their respective distributions (*P*^*sim*^*= 0.006*, *0.004*, <*0.001*, respectively, fig. 3), suggesting natural selection likely drove the change in these allele frequencies. A similar significant shift was observed when we considered DR13 broadly (*P*^*sim*^ < *0.001*). To quantify the selection coefficient (*s*) responsible for these changes, we performed the simulations incorporating selection, mirroring the timeline of the plague, across a range of *s* values. We identified an *s* equal to −0.25 was most likely to produce the observed decrease in B*51:01 alleles as well as an *s* equal to −0.27 in case of C*06:02. An *s* of 0.37 was most likely to cause the increase in DRB1*13:01 (supplementary fig. 5, Supplementary Material online). Notably, these values are within the range of previously reported values of *s* acting on MHC (Radwan et al. 2020).

### Higher Incidence of KIR3DL1 Interaction with HLA-B in Plague Victims Than Modern Inhabitants of Ellwangen

The binding specificity of HLA allotypes, and thus their function and distinctiveness, is determined by specific amino acid residues in the alpha-helix of the molecule. Polymorphism of these amino acid residues is associated with autoimmune diseases and response to pathogens (Achkar et al. 2012; Hammer et al. 2015; Sun et al. 2018; Hollenbach et al. 2019). We identified three of these residues having significant (*P* < 0.05) differences in frequency between the 16th century victims and modern individuals (supplementary data 7A, Supplementary Material online). We observed histidine (H) at position 9 of HLA-DPB1 to be approximately three times more frequent in the 16th century (13%) than the modern (4%) individuals (*P* = 0.03 [*P*-corrected = NS]; DANc = 0.13); supplementary data 7A, Supplementary Material online). We also observed isoleucine (I) at position 80 (I-80) in HLA-B twice as frequently in the 16th century (28%) than in the modern individuals (15%) (*P* = 0.04 [*P*-corrected = NS]; DANc = 0.28), and aspartic acid (D) at position 114 in HLA-C more frequently in the 16th century (85%) than the modern (70%) individuals (*P* = 0.02 [*P*-corrected = NS]; DANc = −0.062); supplementary data 7A, Supplementary Material online). Residue D-114 is located in the outward-facing groove of HLA-C and its variation can directly affect the sequence of endogenous peptides able to bind (Di Marco et al. 2017). Since *HLA-B* alleles encoding I-80 are most commonly observed on haplotypes that also have *HLA-C* alleles that encode D-114 (Cao et al. 2001), it is likely that the observed frequency difference at this position is driven by LD with *HLA-B*. HLA-B*27:02, -B*38:01, -B*49:01, -B*51:01, -B*52:01, -B*57:01, and -B*58:01 are all I-80^+^ allotypes that are more frequent in the 16th century than the modern inhabitants of Ellwangen (supplementary data 7B and 7C, Supplementary Material online), together accounting for the observed difference in I-80 frequency (table 3). Thus, it is likely that the significant difference in frequencies we observed for HLA-B*51:01 can be attributed to the fact that it possesses an isoleucine at position 80. We next tested whether the observed frequency changes in HLA-DPB1 H-9 and HLA-B I-80 were more likely due to genetic drift or natural selection, using neutral forward genetic simulations as above. In both cases, we found these allele frequency shifts were unlikely to be observed unless natural selection was included in the model (HLA-DPB1 H-9 *P*^*sim*^*= 0.014*, HLA-B I-80 *P*^*sim*^*= 0.002*).

*KIR* genes encode surface proteins on natural killer (NK) cells whose interaction with HLA class I molecules can determine the outcome of NK cell responses (Guethlein et al. 2015). For example, polymorphism of residue 80 in HLA-B controls its ability to bind to KIR3DL1, with I-80 defining ligand specificity and permitting the strongest interaction (Saunders et al. 2015). We therefore sought to determine whether the observed high frequency of *HLA-B I-80^+^* alleles in the 16th century samples affects the frequency of HLA-B interaction with KIR3DL1. The *KIR* region varies by gene content (Uhrberg et al. 1997), and we were able to determine this diversity across all the 16th century individuals (supplementary fig. 6, Supplementary Material online). We observed that 97% of the 16th century individuals possess at least one copy of *KIR3DL1*, compared with 88% of the modern Ellwangen samples. These values are within the range observed in modern European populations (Hollenbach et al. 2012) as well as those predicted in our neutral forward genetic simulations (*P*^*sim*^*= 0.258*). Thus, we observe no statistically significant differences in *KIR3DL1* gene frequencies between the 16th century and modern samples (table 4*A*). Similarly, we observed no difference in *KIR3DL1* allele frequencies (supplementary data 6C, Supplementary Material online) between the 16th century and modern individuals (table 4*A*). By contrast, we found that *KIR3DL1 and HLA-B I-80^+^*, and thus their combined genotype, is more frequent in 16th century (53%) than modern (26%) individuals (*P* = 0.011 [*P*-corrected = NS], table 4*B*). Using simulations, we found that genetic drift was unlikely to produce the modern day observed frequencies of *HLA-B I-80 and KIR3DL1*, but that selection against *HLA-B I-80* likely drove the decreased *HLA-B I-80^+^/KIR3DL1^+^* joint genotype frequencies (*P*^*sim*^*= 0.002*).

## Discussion

In this study, we investigate a large panel of immunity-related genes from 36 individuals discovered in three 16th century plague mass graves in Ellwangen, Southern Germany, and compare them with 50 present-day inhabitants of Ellwangen. For this purpose, we developed a targeted DNA capture protocol comprising 488 human immune system genes including the six major *HLA class I and class II* genes and the *KIR* locus. We also compared the 16th century *HLA* allele frequencies with sequence data of 8,862 potential stem cell donors registered with DKMS (German Bone Marrow Donor Registry). Although we observe a predominant genetic stability of human immune genes over at least five centuries in Central Europe, we find distinct allele frequency changes in the *HLA* and in two other genes that encode components of innate immunity.

Given its devastating effect, the *Y. pestis*-driven second plague pandemic is a strong candidate for exerting selection pressure on the human immune response (Lenski 1988; Laayouni et al. 2014). We observed strong allele frequency differences at SNPs located in the *FCN2 and NLRP14* genes, albeit we find no clear evidence that positive selection has contributed to the observed allele frequency differentiation. Both of these molecules are pattern recognition receptors that bind specific pathogen-derived components to initiate the inflammation response; Ficolin-2 does this extracellularly, and NLRP14 intracellularly. Ficolin-2 promotes phagocytosis of pathogenic bacteria (Hoang et al. 2011; Luo et al. 2013). Interestingly, we observed two SNPs of known direct functional effect to be in strong LD, forming a single haplotype that is elevated in frequency in the modern compared with the 16th century individuals. This haplotype both increases serum concentration and alters the binding properties of Ficolin-2 (Hummelshoj et al. 2005; Cedzynski et al. 2007), which makes it a good candidate for providing improved resistance to *Y. pestis* infection. On the other hand, less is known about NLRP14, which has similar domain organization to other inflammasome proteins. Inflammasomes act to trigger inflammation as well as self-destruction of infected cells (Lamkanfi and Dixit 2014) and have been identified recently as important mediators of the immune response to *Y. pestis* (Park et al. 2020). Interestingly, the same variant we observed at lower frequency in modern individuals than plague victims (K-808) was identified at high frequency due to positive selection in the Human Genome Diversity-Project populations from East Asia (Vasseur et al. 2012). Similar inflammasome molecules, including NLRP3 and NLRP12, are known to respond to *Y. pestis* (Vladimer et al. 2012, 2013), but may also be exploited by bacteria to inhibit immunity (Anand et al. 2012; Zaki et al. 2014; Philip et al. 2016). K-808 is located in the LRR domain of NLRP14 and influences ligand specificity. Therefore, the fluctuating frequencies of the variants at this position point to an evolutionary battle between host and pathogen (Abi-Rached et al. 2007). Functional tests are thus required to determine if mutation at residue 808 permits recognition of any components of *Y. pestis*.

On examination of *HLA* alleles, we observed candidates for natural selection of human adaptive immune responses. HLA class I and II are cell surface molecules that bind to peptides derived from intracellular or extracellular proteins, respectively. To trigger and drive the adaptive immune response, these peptides are presented by the HLA molecules to T cells. Antibody production is elicited through highly polymorphic HLA class II molecules, HLA-DP, -DQ, and -DR, presenting pathogen-derived peptides to CD4+ T cells (Neefjes et al. 2011). Direct killing of infected cells can occur when any of three highly polymorphic HLA class I molecules, HLA-A, -B, or -C, presents pathogen-derived peptides to cytotoxic CD8+ T cells (Doherty and Zinkernagel 1975). The HLA-DRB1*13 allelic group increased in frequency from 5.6% in the plague victims to 17% in the modern Ellwangen individuals and 12% in the German bone marrow donors, potentially indicating antibody-driven protection from the plague for individuals having this allotype. HLA-DRB1*13 is associated with resistance to *Mycobacterium tuberculosis* (Dubaniewicz et al. 2000). Similar to *Y. pestis*, *M. tuberculosis* can invade and survive within macrophages (Pieters 2008). Macrophages express high levels of HLA class II and are cells that are specialized for presenting peptides to CD4+ T cells to initiate antibody production. These HLA class II molecules can present antigens from intracellular pathogens, such as *M. tuberculosis* (Ankley et al. 2020). Thus, the same adaptive immune pathway triggered by HLA-DRB1*13 that provides resistance to *M. tuberculosis*, might also provide resistance to *Y. pestis*.

Some HLA class I allotypes interact with killer-cell immunoglobulin-like receptors (KIRs) to modulate the function of NK cells, which are essential components of innate immunity, providing front-line defense against infection (Long et al. 2013; Guethlein et al. 2015). The *KIR* locus varies by gene content (Uhrberg et al. 1997; Wilson et al. 2000) and is located on a separate chromosome (chr19) to HLA (chr6). Combinatorial diversity of HLA class I and KIR allotypes directly impacts NK cell responses to infection (Bashirova et al. 2006; Parham and Moffett 2013). We observed a lower frequency of the HLA-B allotypes that can interact with KIR3DL1 in the modern individuals than we did in the plague victims, suggesting this combination could have been disadvantageous for individuals infected with *Y. pestis*. KIR3DL1 is an inhibitory receptor that enables NK cells to respond strongly to changes in HLA expression by infected cells (Gumperz et al. 1995; Saunders et al. 2015; Boudreau and Hsu 2018). Finally, we observed a marked decrease in the frequency of HLA-C*06:02 when comparing the 16th century and modern Ellwangen populations. HLA-C*06:02, which also interacts strongly with KIR (Hilton et al. 2015), is strongly associated with psoriasis (Ogawa and Okada 2020), an immune-mediated disease. These observations implicate excess collateral damage caused by NK cells responding to infection (Kim et al. 2008; Guo et al. 2018), as a potential mechanism of pathology.

A limitation of this study is the relatively small sample size of 36 plague victims from the 16th century. As suggested by our effect size analyses (supplementary fig. 7, Supplementary Material online), with the given sample size only large effects (*w = *0.40–0.45) can be detected, and therefore, the observed frequency changes do not withstand multiple testing corrections. Thus, it also remains possible that the signals of selection we detected for some variants are caused by drift and/or sampling biases, and, on the other hand, some other variants under selection were potentially not targeted through this approach. Increasing the sample size in future studies will allow addressing this caveat. Moreover, all tested individuals are 16th century late plague victims, and it remains possible that stronger selection signatures could be observed when analyzing individuals who died of plague in earlier pandemics. Importantly, further cohorts of *Y. pestis* victims are required to verify the observations in this study in different geographic contexts, and also whether the associations with the above-mentioned immunity genes are specific to the plague or might be caused by other pathogens (Galvani and Slatkin 2003). Furthermore, the demographic model we used for simulation of natural selection is fitted to the CEU population (Central Europeans from Utah; Gravel et al. 2011) and assumes an exponential population growth. However, the CEU population might have had a different demographic history than Ellwangen. It cannot, therefore, be ruled out that the results from our analysis of natural selection may be inaccurate, if Ellwangen has undergone stronger genetic drift than CEU. Nevertheless, our simulation results provide preliminary evidence for natural selection as the main driving agent for the decrease of frequencies in HLA-C*06:02, HLA-B*51:01 (and other HLA-B I-80^+^ alleles), and HLA-DPB1-H9 on the one hand, and the frequency-increase in HLA-DRB1*13 on the other hand. We note that the frequency changes we observed are based on simulations of episodic selection and could also be derived through alternative scenarios, including constant selection pressure (e.g., *s* of -0.012 B*51, -0.014 C*06, 0.06 DRB1*13; data not shown), or other epidemic challenges, such as smallpox or tuberculosis, occurring since the 16th century. However, we did not find evidence of smallpox or tuberculosis in the plague victims’ DNA. Comparison with nonplague victims from the same time period will be necessary to definitively answer this question. Our results do not provide support for the proposition that the evolution of human immunity drove the reduction of *Y. pestis* virulence and its disappearance from Europe (Ell 1984). Instead, we provide first evidence for evolutionary adaptive processes that might be driven by *Y. pestis* and may have been shaping certain human immunity-relevant genes in Ellwangen and possibly also in Europe. As the earliest victims of *Y. pestis* in Europe were already present in the Late Neolithic (Rasmussen et al. 2015; Andrades Valtuena et al. 2016; Rascovan et al. 2019) and Europeans were intermittently exposed to plague for almost 5,000 years, it is possible that relevant immunity alleles had already been preselected in the European population long ago and maintained by standing variation (Ralph and Coop 2015) but recently became selected through epidemic events. Whilst *Y. pestis* seems a likely culprit, this remains to be determined through replication cohorts and further functional analyses.

## Materials and Methods

### Anthropological Analyses

Anthropological analyses on the skeletal remains were conducted in the Institute of Paleoanthropology, University of Tübingen. Diseases of the periodontium and the teeth, nonspecific stress markers and deficiencies, degenerative transformations, inflammatory bone changes, and trauma were recorded (supplementary note 1, Supplementary material online). The body height of the adult individuals was reconstructed and the growth course of the subadult individuals was analyzed (Kairies 2015).

### C14-Dating of the Archaeological Remains from Ellwangen

Acceleration Mass Spectrometry Radiocarbon (AMS-C14) dating was conducted at the Curt-Engelhorn Center for Archaeometry in Mannheim. Calibration was performed based on the INTCAL13 and the SwissCal 1.0 calibration curves.

### DNA Extraction

Petrous pyramids were cut longitudinally in order to enable access to the bony labyrinth (supplementary fig. 1*C*, Supplementary Material online), which is the densest part of the mammalian body (Frisch et al. 1998) and provides the highest endogenous DNA yields (Pinhasi et al. 2015). After cleaning the surface on one side of the bony labyrinth with the drill bit, sampling was performed along the semicircular canal, which yielded 80- to 120-mg bone powder. DNA extraction was performed by guanidinium–silica-based extraction (Rohland and Hofreiter 2007) using all the bone powder obtained. Tooth samples were cut in the middle, thus separating the crown from the root, followed by drilling into the dental pulp to produce bone powder (ca. 100 mg). Saliva samples were obtained from 51 living Ellwangen citizens using *Whatman OmniSwab* cheek swabs. Samples were obtained only from individuals whose families have been resident in Ellwangen for at least four generations. Consent was given by the contributing persons and their samples were anonymized. Approval for the study was granted by the Ethics Committee of the Faculty of Medicine of the Eberhard Karls University and the University Hospital Tübingen. Isolation of genomic DNA was performed using the *QIAamp DNA Blood Mini Kit* following the *Qiagen* protocol.

### Preparation of Libraries and Sequencing

Overall strategy: Indexed libraries were generated from all samples and the sequencing was then performed in two stages. First, an aliquot of the full DNA library was subjected to whole-genome sequencing. Then, a second aliquot was subjected to enrichment for selected immunity-related genes and subsequently sequenced.

Since our protocols for DNA extraction and library preparation are optimized for short-length ancient DNA, and in order to avoid potential bias through laboratory methods, we sheared the DNA extracted from modern individuals using ultrasonic DNA shearing to the same average length as the ancient DNA. Therefore, the modern DNA was sheared to an average fragment length of 75 bp using a *Covaris M220 Focused ultrasonicator*. DNA libraries, including sample-specific indices, were prepared using 20 µl of each extract following published protocols (Meyer and Kircher 2010; Kircher et al. 2012). For the ancient samples, partial uracil-DNA-glycosylase treatment was first applied (Rohland et al. 2015). Sequencing was performed using an Illumina *Hiseq 4000* instrument with 75 + 8 cycles in single-end (SE) mode.

### Screening for Pathogens

DNA samples were screened for their metagenome content using the alignment tool MALT version 0.3.8 (Vågene et al. 2018) and the metagenome analyzer MEGAN V6.11.4 (Huson et al. 2007) (supplementary table 1, Supplementary Material online).

Since petrous bone samples are not ideal for pathogen screening, we additionally accessed well-preserved tooth samples from 30 distinct 16th century plague victims. Teeth were not available from all individuals from whom we had obtained petrous bones nor could they be unambiguously attributed to specific individuals. Sequencing libraries and shotgun sequencing were performed on the teeth following published protocols as described above.

MALT was used to align all preprocessed reads against a collection of all complete bacterial genomes obtained from NCBI (ftp.ncbi.nlm.nih.gov/genomes/refseq/bacteria, accessed March 12, 2018). MALT was executed in BLASTN mode for bacteria using the following command:

malt-run –mode BlastN –e 0.001 –id 95 –alignmentType SemiGlobal –index $REF –inFile $IN –output $OUT (where $REF is the MALT index). The *e*-value (–e) is a parameter that describes the number of hits that are expected to be found just by chance. The –id parameter describes the minimum percent identity that is needed for a hit to be reported. As the screening with MALT was performed on aDNA data the applied filters are not very stringent since we expect substitutions in organisms from ancient samples.

Reads assigned to the *Y. pestis* node and reads assigned to the nodes below were extracted using the extract reads function in MEGAN. For subsequent verification, blastn (version 2.7.1) was used to blast the extracted reads against *Y. pestis* (NC_003143.1) and *Y. pseudotuberculosis* (NC_010634.1). The following custom blast command was used:

blastn -db $REF -query $IN -outfmt “6 qseqid sseqid pident length mismatch gapopen qstart qend sstart send evalue bitscore gaps” (where $REF is the reference genome and $IN are the extracted reads from MEGAN).

### Targeted Sequencing of Immunity-Related Genes

Indexed libraries containing 20-µl DNA each were amplified in 100-µl reactions in a variable number of one to seven cycles to reach the required concentration of 200 ng/µl for enrichment, followed by purification using *Qiagen MinElute* columns. Using an in-solution capture-by-hybridization approach (Fu et al. 2013), DNA molecule fragments originating from immunity genes were enriched from the total DNA. The design and manufacture of the capture probes are described below. Sequencing was performed as above.

### DNA Damage Estimation

We performed an initial analysis of the merged data using the *EAGER* pipeline (Peltzer et al. 2016) as follows: reads were mapped to *hg19* (The Genome Sequencing Consortium 2001) using the *aln* algorithm in *BWA 0.7.12* (Li and Durbin 2010) with a seed length (k) of 32, the *samtools* mapping quality parameter “q” set to 30 and a reduced mapping stringency parameter “-n 0.01” to account for damage in ancient DNA. On average 2.2 million reads (51%) from the plague victims with an average length of 59 bp, and 4.2 million reads (91%) from the modern individuals with an average length of 68 bp, mapped uniquely to hg19 (supplementary data 1A, Supplementary Material online). To assess the authenticity of the ancient DNA fragments, C to T misincorporation frequencies (Briggs et al. 2007) were obtained using *mapDamage 2.0* (Jonsson et al. 2013). As expected from partial UDG-treatment (Rohland et al. 2015), ancient DNA sequences showed C to T substitutions at the first two positions of their 5ʹ ends and G to A substitutions at the 3ʹ ends (supplementary data 1A, Supplementary Material online). The first two positions from the 5ʹ end of the fastq-reads were trimmed off. The modern sample DNA sequence reads were not subjected to this trimming.

### Sex Determination

Genetic sex was determined based on the ratio of sequences aligning to the X and Y chromosomes compared with the autosomes (Skoglund et al. 2013).

### Final Data Collation

Contamination was estimated through examination of mitochondria sequences using the software *Schmutzi* (Renaud et al. 2015), and in males additionally on the X-chromosomal level by applying *ANGSD* (Korneliussen et al. 2014). Contamination estimates ranged between 1% and 3% on mitochondrial and between 0.2% and 2.9% on X-chromosomal level (supplementary data 1A, Supplementary Material online). Data sets showing >8% contamination were excluded from further analyses.

### Genotyping

SNPs were drawn at random at each position from a previously published data set of 1,233,013 SNPs (Lazaridis et al. 2014; Haak et al. 2015; Mathieson et al. 2015) in a pseudohaploid manner using *pileupcaller* from the *sequenceTools* package (Lamnidis et al. 2018). Samples having fewer than 10,000 calls from a set of 1,233,013 SNPs were excluded. Forty-four data sets from ancient samples (40 from petrous bones and four from teeth) and 52 data sets from modern saliva samples remained.

### Population Genetic Analyses

The genotype data from both Ellwangen populations were merged with a data set of previously published West Eurasian populations genotyped on the aforementioned 1,233,013 SNPs (Mathieson et al. 2015) using the program *mergeit* from the *EIGENSOFT* package (Patterson et al. 2006). PCA was performed using the software *smartpca* (Patterson et al. 2006). Admixture modeling was performed using the software ADMIXTURE (Alexander et al. 2009) with 65 West Eurasian populations from the *Affymetrix Human Origin* data set, and the number of ancestral components ranging from *K* = 3 to *K* = 12. Cross-validation was performed for every admixture model and the model with the highest accuracy was determined by the lowest cross-validation error.

### Kinship Analysis

Kinship was assessed using three different software packages: *READ* (Monroy et al. 2018), *lcMLkin* (Lipatov et al. 2015), and *outgroup f3* statistics (Patterson et al. 2012). *READ* identifies relatives based on the proportion of nonmatching alleles. *lcMLkin* infers individual kinship from calculated genotype likelihoods, and *f3* statistics can be used to identify relatives based on the amount of shared genetic drift. A pair of individuals was regarded related if evidence of relatedness was independently provided by at least two programs . For the modern population, a first-degree relationship (parent–child or siblings) was detected by all three programs for EL1 and EL57. For the plague victims, evidence of a first-degree kinship was provided from all three programs for three pairs of individuals: ELW015 and ELW037, ELW016 and ELW017, and ELW036 and ELW039. Support from at least two programs was given for second-degree relatedness (grandparent–grandchild, uncle–nephew, or first cousins) for two pairs: ELW021 and ELW030, and ELW007 and ELW039. Second- or higher-degree relatedness was suggested for the pair ELW030 and ELW034. Nine further observations of second-degree relationships were observed, but supported by only one program at a time and therefore not regarded as reliable kinship estimates (supplementary fig. 2, Supplementary Material online). Individuals EL57, ELW017, ELW030, ELW037, and ELW039 were excluded from allele frequency calculations, since they constitute kinship “nodes” or “leaves” that would bias allele frequencies as they do not contribute to the total allele diversity.

### Effect Size Analysis

Effect sizes were estimated and plotted in G*Power 3.1.9.2 (Faul et al. 2007) based on the given sample size and a power of 0.8. Effect size analysis has shown that with the current sample size large to medium effects (*w *=* *0.45–0.4) could be detected (supplementary fig. 7, Supplementary Material online).

### Probe Design for Immune-Capture

Enrichment of selected target genomic regions prior to sequencing can save sequencing costs and significantly reduce microbial DNA contaminants (Gnirke et al. 2009; Lazaridis et al. 2014; Haak et al. 2015; Mathieson et al. 2015; Fu et al. 2016). We therefore selected a set of 488 different human genes representative of the innate and adaptive immune system (supplementary table 2, Supplementary Material online). Exon sequences were extracted from the human genome build *hg19* (The Genome Sequencing Consortium 2001) using the *RefSeqGene* records from the *NCBI/Nucleotide* database and then selecting “Highlight Sequence Features” and “Exon.” We added alternative alleles for *HLA*, *MIC*, *TAP*, and *KIR*, which were obtained from the IMGT/HLA database (Robinson et al. 2015). For *HLA class I and KIR* genes the intronic regions were also included. For the *HLA and MIC* genes a set of 83 representative alleles with full-length gene sequences was chosen that encompasses the major serologically defined subclasses (Holdsworth et al. 2009) and covers 95% of the known polymorphism. To capture the remaining 5%, a set of 162 × 160 bp consensus sequences was designed.

A 60-bp probe was designed at every 5-bp interval along the target sequence. The last (3ʹ) 8 bp of each generated sequence was replaced by a custom primer sequence, so that probes could be amplified. The final 52-bp probe sequences were mapped to *hg19* using *RazerS3* (Weese et al. 2012) with minimum threshold of 95% identity. Duplicates and probe sequences that mapped more than 20 times were removed. This process resulted in a final set of 322,667 unique probe sequences of 52-bp length. The probe set was tripled to complete capacity of the *Agilent* 1-million feature array. The probes were cleaved from the array and amplified using PCR (Fu et al. 2016). In summary, we generated 322,667 unique probes of 52-bp length using stepwise 5-bp tiling to cover a total of 3,355,736 bp. The final set of probe sequences is available in supplementary data 8, Supplementary Material online. To validate the capture protocol, we used seven cell lines from the Immunogenetics and Histocompatibility Workshop (IHW) chosen to represent divergent HLA alleles that we had previously sequenced to full resolution (Norman et al. 2017). The results are shown in supplementary data 2, Supplementary Material online.

### Analysis of the CCR5-Δ32 Frequency

The CCR5 locus (chr3:46414947–46414978) was included in the target regions. To genotype CCR5 for wild type (wt) and Δ32 alleles, the sequence data were remapped to *hg19* using BWA-*mem* with the mapping quality filter turned off. To generate genotypes, the CCR5 locus was visually inspected using the Integrative Genomics Viewer (Robinson et al. 2011).

### 1000 Genome Data for Selection Scan

We obtained the “low coverage” and “exome” aligned data for a set of 50 unrelated individuals for the East Asian population CHB (Han Chinese in Beijing, China) from the 1000 Genomes Phase 3 data set (ftp://ftp.1000genomes.ebi.ac.uk/vol1/ftp/phase3/data/, last accessed August 5, 2021). The bam files were converted into *fastq* format using the *bamtofastq* option from the software *bedtools 2.28.0* (Quinlan 2014).

### Variant Detection

We used *samtools mpileup* (Li et al. 2009) for variant detection with a minimum mapping and base quality of 30 while ignoring indels (-q 30 -Q 30 -C 50 -t DP, SP -g –skip-indels), and used bcftools (Li 2011) for variant calling (-m -f GQ -O b). We considered only variants that were within the captured regions ± 1,000 bp. Variants were kept when at least ten individuals had a genotype quality of 30 or higher as measured using vcftools (Danecek et al. 2011). The resulting vcf-files were further annotated by adding the ancestral allele and dbSNP IDs version 147. The ancestral allele was called as the most parsimonious based on 1000 Genomes data (Abecasis et al. 2012) and a multiple species alignment (Key et al. 2016).

### DAnc Calculation

For all variants shared across the Ellwangen data (modern and ancient) and 1000 Genomes CHB population, we calculated a *Differentiation with Ancestral* (*DAnc*) score (Key et al. 2016). *DAnc* is calculated per site and uses DAF estimates to infer population-specific allele frequency changes. Therefore, we inferred the DAF using the annotated ancestral allele for every site. Using the DAF, we calculated *DAnc* scores per site:

$$\mathrm{DAnc}=\left|\left({\mathrm{ELW}}_{\mathrm{MOD}}-\mathrm{CHB}\right)\right|-\left|\left({\mathrm{ELW}}_{\mathrm{ANC}}-\mathrm{CHB}\right)\right|$$

.

For every site, the resulting *DAnc* scores can range from −1 to +1. Invariable sites have a score of 0. A positive *DAnc* score indicates that the modern Ellwangen population has a different allele frequency compared with the ancient Ellwangen population and the outgroup population, for example, due to recent positive selection. A negative *DAnc* score indicates that the ancient Ellwangen population differentiates from both modern Ellwangen and the outgroup CHB.

### Estimating F_ST_ Values

We calculated *F_ST_* values for all variants using the (Weir and Cockerham 1984) estimator implemented in *vcf-tools* (Danecek et al. 2011). We report the empirical *P* values, which were obtained by comparing the *F_ST_* of all three candidate SNPs to the empirical distribution of *F_ST_* scores from all other variants.

### Simulation of Neutral Evolution

In order to estimate the expected distribution of *DAnc* scores under neutral evolution, we simulated the European demographic history, using a published model (Gravel et al. 2011) and the simulation software *slim2* (Haller and Messer 2017). The demographic model is based on genome-wide data; we, however, had predominantly capture data from coding regions. To account for increased drift in coding regions due to background selection, we reduced the effective population sizes using background selection coefficients (*B scores*; Key et al. 2016). We estimated background selection for every genomic region captured using a published genome-wide map (McVicker et al. 2009). The complete model including all parameters is available in supplementary data 9, Supplementary Material online. We ran 100,000 simulations of genomic loci matched in length to the captured region and used the resulting variants to calculate the neutral expectation of the *DAnc* score distribution.

### Simulation of Natural Selection

We performed 10,000 forward genetic simulations using *slim3* (Haller and Messer 2019) to determine null distributions for neutral frequency changes over 500 years in Ellwangen for each HLA and KIR allotype. We used the sampled ancestral Ellwangen HLA and KIR allotype frequencies as input and simulated 20 generations, assuming a 25 year generation time (Gravel et al. 2011). We assumed a constant population growth rate of 1.085 per generation, resulting in growth from 5,000 to approximately 25,000 in Ellwangen. We explicitly modeled HLA and KIR allotypes, using linked binary identifiers to differentiate between alleles of a gene, and therefore assumed no new mutations or intragenic recombination. We calculated intergenic recombination rates per generation between *HLA* genes using a recent sex-averaged refined genetic map (Bhérer et al. 2017). We allowed free recombination between *HLA and KIR* regions (because they are on separate chromosomes). Specifically, we assumed the following number of crossovers per generation between each HLA gene. *HLA-A*/*HLA-C*: 5.3e^−3^, *HLA-C*/*HLA-B*: 1e^−8^, *HLA-B*/*HLA-DRB345*: 5.4e^−3^, *HLA-DRB345*/*HLA-DRB1*: 1e^−8^, *HLA-DRB1*/*HLA-DQA1*: 3.2e^−5^, *HLA-DQA1*/*HLA-DQB1*: 3.9e^−7^, *HLA-DQB1*/*HLA-DPA1*: 6.5e^−3^, and *HLA-DPA1*/*HLA-DPB1*: 1e^−8^. We calculated neutral frequency changes of each allotype. We conclude the frequency changes of an allotype are due to natural selection if the sampled modern-day allotype frequency falls within the 0.5% extremes of the respective neutral distribution (*P* < 0.01).

We reimplemented the neutral slim3 models as described above, but included a nonzero *s* parameter for each HLA-allotype in question. For each selected allotype, we ran 100 simulations with a positive or negative *s* with an absolute value of 0.001, 0.01, 0.1, 0.2, 0.3, 0.4, 0.5, 0.6, or 0.7. Mirroring the timeline of the European plague outbreak, allotypes were selected for seven generations and then returned to neutrality. The reported *s* values were consistent with previous reported values of *s* acting on *MHC* genes (Radwan et al. 2020). We estimated the strength of natural selection by fitting a LOESS curve to the simulated relationship between *s* and allotype frequency and mapping the observed modern Ellwangen allotype frequency.

### HLA Typing of the Ellwangen Individuals

We applied the *OptiType* algorithm, which is a program that enables HLA genotyping from high-throughput sequence data. *OptiType* requires a minimum of a 12-fold coverage to reliably determine the *HLA* alleles present at two-field (distinct polypeptide sequences) resolution. We applied *OptiType* (Szolek et al. 2014) to identify *HLA class I* alleles, using a reference set of present-day *HLA* allele sequences and a required sequence identity of at least 97% for every alignment. We set no limit on the number of potential best matches during read mapping. We manually verified the results obtained by a development version of the upcoming OptiType 2.0 package in order to determine *HLA class II* alleles. For every sample, the *OptiType* call having highest confidence was used.

### Reconstruction of HLA Haplotypes

Haplotypes were assigned based on previously reported frequencies and LD (Cao et al. 2001; González-Neira et al. 2004). Maximum-likelihood haplotype frequencies for alleles and two-point, three-point, and four-point associations were estimated using an Expectation-Maximization (EM) algorithm provided by the computer program *Arlequin* ver. 3.5 (Excoffier and Lischer 2010).

### Comparing Allele and Haplotype Frequencies

*HLA* allele frequencies were calculated from *HLA-A*, *-B*, *-C*, and *-DRB1* sequence data of 8,862 potential stem cell donors registered with DKMS (German Bone Marrow Donor Registry) by June 2014. Donors were of self-assessed German origin. Allele frequencies were calculated to the two-field level (polypeptide sequence; Schmidt et al. 2009). For allele frequency comparisons, chi-square tests (Pearson 1900) were applied in R (R Development Core Team 2011). Pairwise proportion tests were made between the allele or haplotype frequencies, where *P *<* *0.05 was considered significant. Omnibus tests for association with specific amino acid positions, as well as pairwise tests for specific residues, were computed using the BIGDAWG R package (Pappas et al. 2016). Linear mixed-effects modeling was performed using the Gaston package in R (https://cran.r-project.org/web/packages/gaston/, last accessed August 5, 2021) and the *lcMLkin* kinship matrix generated as above. Wilson Score Interval estimation was performed using the “Hmisc” package of R (https://CRAN.R-project.org/package=Hmisc, last accessed August 5, 2021).

### Reconstruction of KIR Genotypes and KIR Allele Frequency Analyses

Sequence reads specific to the *KIR* locus were identified by alignment to the human genome reference hg19 using BWA *mem*, and then extracting those mapping to chr19:55,228,188–55,383,188 or chr19_gl000209_random. The presence or absence and copy number of each *KIR* gene were determined using the PING pipeline (Norman et al. 2016), modified for SE (i.e., nonpaired) reads. The alleles of *KIR3DL1/S1* were also determined using an SE modified version of PING and further validated by determining the alleles of the genes flanking *KIR3DL1/S1* in the telomeric portion of the *KIR* locus. As an additional step, virtual sequence probes were used to identify specific alleles directly from FASTQ data files, with a threshold of ten reads used to positively identify a given allele. The scripts and probe sequences are available at https://github.com/n0rmski/ThePlague/.

## Supplementary Material

Supplementary data are available at *Molecular Biology and Evolution* online.

## Supplementary Material

msab147_Supplementary_Data

## References

[msab147-B1] Abecasis GR , Auton A, Brooks LD, DePristo MA, Durbin RM, Handsaker RE, Kang HM, Marth GT, McVean GA; 1000 Genomes Project Consortium. 2012. An integrated map of genetic variation from 1,092 human genomes. Nature 491(7422):56–65. 23128226 10.1038/nature11632 PMC3498066

[msab147-B2] Abi-Rached L , Dorighi K, Norman PJ, Yawata M, Parham P. 2007. Episodes of natural selection shaped the interactions of IgA-Fc with FcalphaRI and bacterial decoy proteins. J Immunol. 178(12):7943–7954. 17548632 10.4049/jimmunol.178.12.7943

[msab147-B3] Achkar J-P , Klei L, de Bakker PIW, Bellone G, Rebert N, Scott R, Lu Y, Regueiro M, Brzezinski A, Kamboh MI, et al 2012. Amino acid position 11 of HLA-DRβ1 is a major determinant of chromosome 6p association with ulcerative colitis. Genes Immun. 13(3):245–252. 22170232 10.1038/gene.2011.79 PMC3341846

[msab147-B4] Al Nabhani Z , Dietrich G, Hugot JP, Barreau F. 2017. Nod2: the intestinal gate keeper. PLoS Pathog. 13(3):e1006177. 28253332 10.1371/journal.ppat.1006177 PMC5333895

[msab147-B5] Albright FS , Orlando P, Pavia AT, Jackson GG, Cannon Albright LA. 2008. Evidence for a heritable predisposition to death due to influenza. J Infect Dis. 197(1):18–24. 18171280 10.1086/524064

[msab147-B6] Alexander DH , Novembre J, Lange K. 2009. Fast model-based estimation of ancestry in unrelated individuals. Genome Res. 19(9):1655–1664. 19648217 10.1101/gr.094052.109 PMC2752134

[msab147-B7] Alfirevic A , Gonzalez-Galarza F, Bell C, Martinsson K, Platt V, Bretland G, Evely J, Lichtenfels M, Cederbrant K, French N, et al 2012. In silico analysis of HLA associations with drug-induced liver injury: use of a HLA-genotyped DNA archive from healthy volunteers. Genome Med. 4(6):51. 22732016 10.1186/gm350 PMC3698530

[msab147-B8] Anand PK , Malireddi RK, Lukens JR, Vogel P, Bertin J, Lamkanfi M, Kanneganti TD. 2012. NLRP6 negatively regulates innate immunity and host defence against bacterial pathogens. Nature 488(7411):389–393. 22763455 10.1038/nature11250 PMC3422416

[msab147-B9] Andrades Valtuena A , Mittnik A, Key FM, Haak W, Allmäe R, Belinskij A, Daubaras M, Feldman M, Jankauskas R, Janković I. 2016. The Stone Age plague: 1000 years of persistence in Eurasia. *Curr Biol. *27:3683–3691. 10.1016/j.cub.2017.10.025 29174893

[msab147-B10] Ankley L , Thomas S, Olive AJ. 2020. Fighting persistence: how chronic infections with *Mycobacterium tuberculosis* evade T cell-mediated clearance and new strategies to defeat them. Infect Immun. 88:e00916-19. 32094248 10.1128/IAI.00916-19 PMC7309621

[msab147-B11] Barreiro LB , Ben-Ali M, Quach H, Laval G, Patin E, Pickrell JK, Bouchier C, Tichit M, Neyrolles O, Gicquel B, et al 2009. Evolutionary dynamics of human Toll-like receptors and their different contributions to host defense. PLoS Genet. 5(7):e1000562. 19609346 10.1371/journal.pgen.1000562 PMC2702086

[msab147-B12] Bashirova AA , Martin MP, McVicar DW, Carrington M. 2006. The killer immunoglobulin-like receptor gene cluster: tuning the genome for defense. Annu Rev Genomics Hum Genet. 7:277–300. 16824023 10.1146/annurev.genom.7.080505.115726

[msab147-B13] Benedictow OJ. 2004. The Black Death, 1346-1353: The Complete History. Vol. 383. Woodbridge, Suffolk, UK: Boydell & Brewer.

[msab147-B14] Bhérer C , Campbell CL, Auton A. 2017. Refined genetic maps reveal sexual dimorphism in human meiotic recombination at multiple scales. Nat Commun. 8(1):14994. 28440270 10.1038/ncomms14994 PMC5414043

[msab147-B15] Biraben J-N. 1976. Les hommes et la peste en France et dans les pays européens et méditerranéens. Mouton: Paris-La Haye.

[msab147-B16] Bjorkman PJ , Parham P. 1990. Structure, function, and diversity of class I major histocompatibility complex molecules. Annu Rev Biochem. 59(1):253–288. 2115762 10.1146/annurev.bi.59.070190.001345

[msab147-B17] Bos KI , Herbig A, Sahl J, Waglechner N, Fourment M, Forrest SA, Klunk J, Schuenemann VJ, Poinar D, Kuch M, et al 2016. Eighteenth century *Yersinia pestis* genomes reveal the long-term persistence of an historical plague focus. Elife 5:e12994. 10.7554/eLife.12994 PMC4798955 26795402

[msab147-B18] Bos KI , Schuenemann VJ, Golding GB, Burbano HA, Waglechner N, Coombes BK, McPhee JB, DeWitte SN, Meyer M, Schmedes S, et al 2011. A draft genome of *Yersinia pestis* from victims of the Black Death. Nature 478(7370):506–510. 21993626 10.1038/nature10549 PMC3690193

[msab147-B19] Boudreau JE , Hsu KC. 2018. Natural killer cell education and the response to infection and cancer therapy: stay tuned. Trends Immunol. 39(3):222–239. 29397297 10.1016/j.it.2017.12.001 PMC6013060

[msab147-B20] Bowsky WM. 1971. The Black Death: a turning point in history? New York: HOlt, Rinhart and Winston.

[msab147-B21] Briggs AW , Stenzel U, Johnson PLF, Green RE, Kelso J, Prüfer K, Meyer M, Krause J, Ronan MT, Lachmann M, et al 2007. Patterns of damage in genomic DNA sequences from a Neandertal. Proc Natl Acad Sci U S A. 104(37):14616–14621. 17715061 10.1073/pnas.0704665104 PMC1976210

[msab147-B22] Büntgen U , Ginzler C, Esper J, Tegel W, McMichael AJ. 2012. Digitizing historical plague. Clin Infect Dis. 55(11):1586–1588. 22918996 10.1093/cid/cis724 PMC3491856

[msab147-B23] Cao K , Hollenbach J, Shi X, Shi W, Chopek M, Fernández-Viña MA. 2001. Analysis of the frequencies of HLA-A, B, and C alleles and haplotypes in the five major ethnic groups of the United States reveals high levels of diversity in these loci and contrasting distribution patterns in these populations. Hum Immunol. 62(9):1009–1030. 11543903 10.1016/s0198-8859(01)00298-1

[msab147-B24] Cedzynski M , Nuytinck L, Atkinson APM, St Swierzko A, Zeman K, Szemraj J, Szala A, Turner ML, Kilpatrick DC. 2007. Extremes of L-ficolin concentration in children with recurrent infections are associated with single nucleotide polymorphisms in the FCN2 gene. Clin Exp Immunol. 150(1):99–104. 17680820 10.1111/j.1365-2249.2007.03471.x PMC2219292

[msab147-B25] Clouse M. 2002. The Black Death transformed: disease and culture in early renaissance Europe: Samuel K Cohn Jr. London and New York: Arnold and Oxford University Press, 2002, pp. 318, US$65.00 (HB). Int J Epidemiol. 31(6):1280–1281.

[msab147-B26] Cohn SK Jr. 2003. The Black Death transformed: disease and culture in early renaissance Europe. London: Edward Arnold. 15043056

[msab147-B27] Danecek P , Auton A, Abecasis G, Albers CA, Banks E, DePristo MA, Handsaker RE, Lunter G, Marth GT, Sherry ST, et al; 1000 Genomes Project Analysis Group. 2011. The variant call format and VCFtools. Bioinformatics 27(15):2156–2158. 21653522 10.1093/bioinformatics/btr330 PMC3137218

[msab147-B28] Darke C , Guttridge MG, Thompson J, McNamara S, Street J, Thomas M. 1998. HLA class I (A, B) and II (DR, DQ) gene and haplotype frequencies in blood donors from Wales. Exp Clin Immunogenet. 15(2):69–83. 9691201 10.1159/000019057

[msab147-B29] Dean M , Carrington M, Winkler C, Huttley GA, Smith MW, Allikmets R, Goedert JJ, Buchbinder SP, Vittinghoff E, Gomperts E, et al 1996. Genetic restriction of HIV-1 infection and progression to AIDS by a deletion allele of the CKR5 structural gene. Hemophilia Growth and Development Study, Multicenter AIDS Cohort Study, Multicenter Hemophilia Cohort Study, San Francisco City Cohort, ALIVE Study. Science 273(5283):1856–1862. 8791590 10.1126/science.273.5283.1856

[msab147-B30] Di Marco M , Schuster H, Backert L, Ghosh M, Rammensee HG, Stevanovic S. 2017. Unveiling the peptide motifs of HLA-C and HLA-G from naturally presented peptides and generation of binding prediction matrices. J Immunol. 199(8):2639–2651. 28904123 10.4049/jimmunol.1700938

[msab147-B31] Doherty PC , Zinkernagel RM. 1975. A biological role for the major histocompatibility antigens. Lancet 1(7922):1406–1409. 49564 10.1016/s0140-6736(75)92610-0

[msab147-B32] Drummond WK , Nelson CA, Fowler J, Epson EE, Mead PS, Lawaczeck EW. 2014. Plague in a pediatric patient: case report and use of polymerase chain reaction as a diagnostic aid. J Pediatric Infect Dis Soc. 3(4):e38–e41. 26625461 10.1093/jpids/piu001

[msab147-B33] Dubaniewicz A , Lewko B, Moszkowska G, Zamorska B, Stepinski J. 2000. Molecular subtypes of the HLA-DR antigens in pulmonary tuberculosis. Int J Infect Dis. 4(3):129–133. 11179915 10.1016/s1201-9712(00)90073-0

[msab147-B34] Dunne C , Crowley J, Hagan R, Rooney G, Lawlor E. 2008. HLA-A, B, Cw, DRB1, DQB1 and DPB1 alleles and haplotypes in the genetically homogenous Irish population. Int J Immunogenet. 35(4–5):295–302. 18976432 10.1111/j.1744-313X.2008.00779.x

[msab147-B35] Ell SR. 1984. Immunity as a factor in the epidemiology of medieval plague. Rev Infect Dis. 6(6):866–879. 6395271 10.1093/clinids/6.6.866

[msab147-B36] Ellwangen S. 2007. Die dunkle Zeit Hexenverfolgung in der Stadt und Fürstpropstei Ellwangen. Ellwangen: Stadtverwaltung Ellwangen.

[msab147-B37] Everitt AR , Clare S, Pertel T, John SP, Wash RS, Smith SE, Chin CR, Feeley EM, Sims JS, Adams DJ, et al; MOSAIC Investigators. 2012. IFITM3 restricts the morbidity and mortality associated with influenza. Nature 484(7395):519–523. 22446628 10.1038/nature10921 PMC3648786

[msab147-B38] Excoffier L , Lischer HEL. 2010. Arlequin suite ver 3.5: a new series of programs to perform population genetics analyses under Linux and Windows. Mol Ecol Resour. 10(3):564–567. 21565059 10.1111/j.1755-0998.2010.02847.x

[msab147-B39] *Faul F, Erdfelder E, Lang A-G, Buchner A. 2007. GPower 3: a flexible statistical power analysis program for the social, behavioral, and biomedical sciences. Behav Res Methods. 39(2):175–191. 17695343 10.3758/bf03193146

[msab147-B40] Feldman M , Harbeck M, Keller M, Spyrou MA, Rott A, Trautmann B, Scholz HC, Päffgen B, Peters J, McCormick M, et al 2016. A high-coverage *Yersinia pestis* genome from a sixth-century justinianic plague victim. Mol Biol Evol. 33(11):2911–2923. 27578768 10.1093/molbev/msw170 PMC5062324

[msab147-B41] Frisch T , Sorensen MS, Overgaard S, Lind M, Bretlau P. 1998. Volume-referent bone turnover estimated from the interlabel area fraction after sequential labeling. Bone 22(6):677–682. 9626408 10.1016/s8756-3282(98)00050-7

[msab147-B42] Fu Q , Meyer M, Gao X, Stenzel U, Burbano HA, Kelso J, Pääbo S. 2013. DNA analysis of an early modern human from Tianyuan Cave. Proc Natl Acad Sci U S A. 110(6):2223–2227. 23341637 10.1073/pnas.1221359110 PMC3568306

[msab147-B43] Fu Q , Posth C, Hajdinjak M, Petr M, Mallick S, Fernandes D, Furtwängler A, Haak W, Meyer M, Mittnik A, et al 2016. The genetic history of Ice Age Europe. Nature 534(7606):200–205. 27135931 10.1038/nature17993 PMC4943878

[msab147-B44] Galvani AP , Slatkin M. 2003. Evaluating plague and smallpox as historical selective pressures for the CCR5-Delta 32 HIV-resistance allele. Proc Natl Acad Sci U S A. 100(25):15276–15279. 14645720 10.1073/pnas.2435085100 PMC299980

[msab147-B45] Gnirke A , Melnikov A, Maguire J, Rogov P, LeProust EM, Brockman W, Fennell T, Giannoukos G, Fisher S, Russ C, et al 2009. Solution hybrid selection with ultra-long oligonucleotides for massively parallel targeted sequencing. Nat Biotechnol. 27(2):182–189. 19182786 10.1038/nbt.1523 PMC2663421

[msab147-B46] González-Neira A , Calafell F, Navarro A, Lao O, Cann H, Comas D, Bertranpetit J. 2004. Geographic stratification of linkage disequilibrium: a worldwide population study in a region of chromosome 22. Hum Genomics. 1(6):399–409. 15606995 10.1186/1479-7364-1-6-399 PMC3500194

[msab147-B47] Gravel S , Henn BM, Gutenkunst RN, Indap AR, Marth GT, Clark AG, Yu F, Gibbs RA, Bustamante CD; 1000 Genomes Project. 2011. Demographic history and rare allele sharing among human populations. Proc Natl Acad Sci U S A. 108(29):11983–11988. 21730125 10.1073/pnas.1019276108 PMC3142009

[msab147-B48] Guethlein LA , Norman PJ, Hilton HG, Parham P. 2015. Co-evolution of MHC class I and variable NK cell receptors in placental mammals. Immunol Rev. 267(1):259–282. 26284483 10.1111/imr.12326 PMC4587382

[msab147-B49] Gumperz JE , Litwin V, Phillips JH, Lanier LL, Parham P. 1995. The Bw4 public epitope of HLA-B molecules confers reactivity with natural killer cell clones that express NKB1, a putative HLA receptor. J Exp Med. 181(3):1133–1144. 7532677 10.1084/jem.181.3.1133 PMC2191933

[msab147-B50] Guo Y , Patil NK, Luan L, Bohannon JK, Sherwood ER. 2018. The biology of natural killer cells during sepsis. Immunology 153(2):190–202. 29064085 10.1111/imm.12854 PMC5765373

[msab147-B51] Haak W , Lazaridis I, Patterson N, Rohland N, Mallick S, Llamas B, Brandt G, Nordenfelt S, Harney E, Stewardson K, et al 2015. Massive migration from the steppe was a source for Indo-European languages in Europe. Nature 522(7555):207–211. 25731166 10.1038/nature14317 PMC5048219

[msab147-B52] Haller BC , Messer PW. 2017. SLiM 2: flexible, interactive forward genetic simulations. Mol Biol Evol. 34(1):230–240. 27702775 10.1093/molbev/msw211

[msab147-B53] Haller BC , Messer PW. 2019. SLiM 3: forward genetic simulations beyond the Wright–Fisher model. Mol Biol Evol. 36(3):632–637. 30517680 10.1093/molbev/msy228 PMC6389312

[msab147-B54] Hammer C , Begemann M, McLaren PJ, Bartha I, Michel A, Klose B, Schmitt C, Waterboer T, Pawlita M, Schulz TF, et al 2015. Amino acid variation in HLA class II proteins is a major determinant of humoral response to common viruses. Am J Hum Genet. 97(5):738–743. 26456283 10.1016/j.ajhg.2015.09.008 PMC4667104

[msab147-B55] Harrison GF , Sanz J, Boulais J, Mina MJ, Grenier J-C, Leng Y, Dumaine A, Yotova V, Bergey CM, Nsobya SL, et al 2019. Natural selection contributed to immunological differences between hunter-gatherers and agriculturalists. Nat Ecol Evol. 3(8):1253–1264. 31358949 10.1038/s41559-019-0947-6 PMC6684323

[msab147-B56] Hellenthal G , Busby GB, Band G, Wilson JF, Capelli C, Falush D, Myers S. 2014. A genetic atlas of human admixture history. Science 343(6172):747–751. 24531965 10.1126/science.1243518 PMC4209567

[msab147-B57] Hilton HG , Guethlein LA, Goyos AA-OX, Nemat-Gorgani N, Bushnell DA, Norman PJ, Parham P. 2015. Polymorphic HLA-C receptors balance the functional characteristics of KIR haplotypes. J Immunol. 195:3160–31770. 26311903 10.4049/jimmunol.1501358 PMC4575877

[msab147-B58] Hoang HV , Toan NL, Song LH, Ouf EA, Bock C-T, Kremsner PG, Kun JFJ, TP V. 2011. Ficolin-2 levels and FCN2 haplotypes influence hepatitis B infection outcome in Vietnamese patients. PLoS One 6(11):e28113. 22140517 10.1371/journal.pone.0028113 PMC3222672

[msab147-B59] Holdsworth R , Hurley CK, Marsh SGE, Lau M, Noreen HJ, Kempenich JH, Setterholm M, Maiers M. 2009. The HLA dictionary 2008: a summary of HLA-A, -B, -C, -DRB1/3/4/5, and -DQB1 alleles and their association with serologically defined HLA-A, -B, -C, -DR, and -DQ antigens. Tissue Antigens 73(2):95–170. 19140825 10.1111/j.1399-0039.2008.01183.x

[msab147-B60] Hollenbach J , Nocedal I, Ladner MB, Single RM, Trachtenberg EA. 2012. Killer cell immunoglobulin-like receptor (KIR) gene content variation in the HGDP-CEPH populations. Immunogenetics 64(10):719–737. 22752190 10.1007/s00251-012-0629-x PMC3438391

[msab147-B61] Hollenbach JA , Norman PJ, Creary LE, Damotte V, Montero-Martin G, Caillier S, Anderson KM, Misra MK, Nemat-Gorgani N, Osoegawa K, et al 2019. A specific amino acid motif of HLA-DRB1 mediates risk and interacts with smoking history in Parkinson's disease. Proc Natl Acad Sci U S A. 116(15):7419–7424. 30910980 10.1073/pnas.1821778116 PMC6462083

[msab147-B62] Hummelshoj T , Munthe-Fog L, Madsen HO, Fujita T, Matsushita M, Garred P. 2005. Polymorphisms in the FCN2 gene determine serum variation and function of Ficolin-2. Hum Mol Genet. 14(12):1651–1658. 15879437 10.1093/hmg/ddi173

[msab147-B63] Huson DH , Auch AF, Qi J, Schuster SC. 2007. MEGAN analysis of metagenomic data. Genome Res. 17(3):377–386. 17255551 10.1101/gr.5969107 PMC1800929

[msab147-B64] Inohara C , McDonald C, Nunez G. 2005. NOD-LRR proteins: role in host-microbial interactions and inflammatory disease. Annu Rev Biochem. 74:355–383. 15952891 10.1146/annurev.biochem.74.082803.133347

[msab147-B65] Johansson Å , Ingman M, Mack SJ, Erlich H, Gyllensten U. 2008. Genetic origin of the Swedish Sami inferred from HLA class I and class II allele frequencies. Eur J Hum Genet. 16(11):1341–1349. 18478041 10.1038/ejhg.2008.88

[msab147-B66] Jonsson H , Ginolhac A, Schubert M, Johnson PL, Orlando L. 2013. mapDamage2.0: fast approximate Bayesian estimates of ancient DNA damage parameters. Bioinformatics 29(13):1682–1684. 23613487 10.1093/bioinformatics/btt193 PMC3694634

[msab147-B67] Kairies M-S. 2015. Drei frühneuzeitliche Massengräber aus Ellwangen (Jagst)—Paläopathologie und demographische Struktur. Master of Science, University of Tübingen, Germany.

[msab147-B68] Karlsson EK , Harris JB, Tabrizi S, Rahman A, Shlyakhter I, Patterson N, O'Dushlaine C, Schaffner SF, Gupta S, Chowdhury F, et al 2013. Natural selection in a bangladeshi population from the cholera-endemic ganges river delta. *Sci Transl Med*. 5:192ra86. 10.1126/scitranslmed.3006338 PMC4367964 23825302

[msab147-B69] Keller M , Spyrou MA, Scheib CL, Neumann GU, Kröpelin A, Haas-Gebhard B, Päffgen B, Haberstroh J, Ribera I Lacomba A, Raynaud C, et al 2019. Ancient genomes from across Western Europe reveal early diversification during the First Pandemic (541-750). Proc Natl Acad Sci U S A. 116(25):12363–12372. 31164419 10.1073/pnas.1820447116 PMC6589673

[msab147-B70] Key FM , Fu Q, Romagné F, Lachmann M, Andrés AM. 2016. Human adaptation and population differentiation in the light of ancient genomes. Nat Commun. 7:10775. 26988143 10.1038/ncomms10775 PMC4802047

[msab147-B71] Key FM , Peter B, Dennis MY, Huerta-Sánchez E, Tang W, Prokunina-Olsson L, Nielsen R, Andrés AM. 2014. Selection on a variant associated with improved viral clearance drives local, adaptive pseudogenization of interferon lambda 4 (IFNL4). *PLoS Genet*. 10(10):e1004681. 10.1371/journal.pgen.1004681 PMC4199494 25329461

[msab147-B72] Kim S , Sunwoo JB, Yang L, Choi T, Song Y-J, French AR, Vlahiotis A, Piccirillo JF, Cella M, Colonna M, et al 2008. HLA alleles determine differences in human natural killer cell responsiveness and potency. Proc Natl Acad Sci U S A. 105(8):3053–3058. 18287063 10.1073/pnas.0712229105 PMC2268583

[msab147-B73] Kircher M , Sawyer S, Meyer M. 2012. Double indexing overcomes inaccuracies in multiplex sequencing on the Illumina platform. Nucleic Acids Res. 40(1):e3. 22021376 10.1093/nar/gkr771 PMC3245947

[msab147-B74] Klebanov N. 2018. Genetic predisposition to infectious disease. Cureus 10(8):e3210. 30405986 10.7759/cureus.3210 PMC6205876

[msab147-B75] Korneliussen TS , Albrechtsen A, Nielsen R. 2014. ANGSD: analysis of next generation sequencing data. BMC Bioinformatics 15:356. 25420514 10.1186/s12859-014-0356-4 PMC4248462

[msab147-B76] Kwiatkowski DP. 2005. How malaria has affected the human genome and what human genetics can teach us about malaria. Am J Hum Genet. 77(2):171–192. 16001361 10.1086/432519 PMC1224522

[msab147-B77] Laayouni H , Oosting M, Luisi P, Ioana M, Alonso S, Ricaño-Ponce I, Trynka G, Zhernakova A, Plantinga TS, Cheng S-C, et al 2014. Convergent evolution in European and Rroma populations reveals pressure exerted by plague on Toll-like receptors. Proc Natl Acad Sci U S A. 111(7):2668–2673. 24550294 10.1073/pnas.1317723111 PMC3932890

[msab147-B78] Lamkanfi M , Dixit VM. 2014. Mechanisms and functions of inflammasomes. Cell 157(5):1013–1022. 24855941 10.1016/j.cell.2014.04.007

[msab147-B79] Lamnidis TC , Majander K, Jeong C, Salmela E, Wessman A, Moiseyev V, Khartanovich V, Balanovsky O, Ongyerth M, Weihmann A, et al 2018. Ancient Fennoscandian genomes reveal origin and spread of Siberian ancestry in Europe. Nat Commun. 9(1):5018. 30479341 10.1038/s41467-018-07483-5 PMC6258758

[msab147-B80] Lazaridis I , Patterson N, Mittnik A, Renaud G, Mallick S, Kirsanow K, Sudmant PH, Schraiber JG, Castellano S, Lipson M, et al 2014. Ancient human genomes suggest three ancestral populations for present-day Europeans. Nature 513(7518):409–413. 25230663 10.1038/nature13673 PMC4170574

[msab147-B81] Lenski RE. 1988. Evolution of plague virulence. Nature 334(6182):473–474. 3405295 10.1038/334473a0

[msab147-B82] Li H. 2011. A statistical framework for SNP calling, mutation discovery, association mapping and population genetical parameter estimation from sequencing data. Bioinformatics 27(21):2987–2993. 21903627 10.1093/bioinformatics/btr509 PMC3198575

[msab147-B83] Li H , Durbin R. 2010. Fast and accurate long-read alignment with Burrows-Wheeler transform. Bioinformatics 26(5):589–595. 20080505 10.1093/bioinformatics/btp698 PMC2828108

[msab147-B84] Li H , Handsaker B, Wysoker A, Fennell T, Ruan J, Homer N, Marth G, Abecasis G, Durbin R; Genome Project Data Processing Subgroup. 2009. The Sequence Alignment/Map format and SAMtools. Bioinformatics 25(16):2078–2079. 19505943 10.1093/bioinformatics/btp352 PMC2723002

[msab147-B85] Lindo J , Huerta-Sánchez E, Nakagome S, Rasmussen M, Petzelt B, Mitchell J, Cybulski JS, Willerslev E, DeGiorgio M, Malhi RS. 2016. A time transect of exomes from a Native American population before and after European contact. Nat Commun. 7:13175. 27845766 10.1038/ncomms13175 PMC5116069

[msab147-B86] Lipatov M , Sanjeev K, Patro R, Veeramah K. 2015. Maximum likelihood estimation of biological relatedness from low coverage sequencing data. *bioRxiv*. 10.1101/023374

[msab147-B87] Long EO , Kim HS, Liu D, Peterson ME, Rajagopalan S. 2013. Controlling natural killer cell responses: integration of signals for activation and inhibition. Annu Rev Immunol. 31:227–258. 23516982 10.1146/annurev-immunol-020711-075005 PMC3868343

[msab147-B88] Luo F , Sun X, Wang Y, Wang Q, Wu Y, Pan Q, Fang C, Zhang X-L. 2013. Ficolin-2 defends against virulent Mycobacteria tuberculosis infection in vivo, and its insufficiency is associated with infection in humans. PLoS One 8(9):e73859. 24040095 10.1371/journal.pone.0073859 PMC3767610

[msab147-B89] Martinon F , Burns K, Tschopp J. 2002. The inflammasome: a molecular platform triggering activation of inflammatory caspases and processing of proIL-beta. Mol Cell. 10(2):417–426. 12191486 10.1016/s1097-2765(02)00599-3

[msab147-B90] Mathieson I , Lazaridis I, Rohland N, Mallick S, Patterson N, Roodenberg SA, Harney E, Stewardson K, Fernandes D, Novak M, et al 2015. Genome-wide patterns of selection in 230 ancient Eurasians. Nature 528(7583):499–503. 26595274 10.1038/nature16152 PMC4918750

[msab147-B91] McManus KA-O , Taravella AM, Henn BA-OX, Bustamante CD, Sikora M, Cornejo OA-O. 2017. Population genetic analysis of the DARC locus (Duffy) reveals adaptation from standing variation associated with malaria resistance in humans. PLoS Genet. 13(3):e1006560. 28282382 10.1371/journal.pgen.1006560 PMC5365118

[msab147-B92] McVicker G , Gordon D, Davis C, Green P. 2009. Widespread genomic signatures of natural selection in hominid evolution. PLoS Genet. 5(5):e1000471. 19424416 10.1371/journal.pgen.1000471 PMC2669884

[msab147-B93] Meyer M , Kircher M. 2010. Illumina sequencing library preparation for highly multiplexed target capture and sequencing. Cold Spring Harb Protoc. 2010(6):pdb.prot5448. 20516186 10.1101/pdb.prot5448

[msab147-B94] Monroy K , Jose M, Jakobsson M, Günther T. 2018. Estimating genetic Kin Relationships in prehistoric Populations. *PlosOne* 13:e0195491. 10.1371/journal.pone.0195491 PMC5912749 29684051

[msab147-B95] Morelli G , Song Y, Mazzoni CJ, Eppinger M, Roumagnac P, Wagner DM, Feldkamp M, Kusecek B, Vogler AJ, Li Y, et al 2010. *Yersinia pestis* genome sequencing identifies patterns of global phylogenetic diversity. Nat Genet. 42(12):1140–1143. 21037571 10.1038/ng.705 PMC2999892

[msab147-B96] Namouchi A , Guellil M, Kersten O, Hänsch S, Ottoni C, Schmid BV, Pacciani E, Quaglia L, Vermunt M, Bauer EL, et al 2018. Integrative approach using *Yersinia pestis* genomes to revisit the historical landscape of plague during the Medieval Period. Proc Natl Acad Sci U S A. 115(50):E11790–E11797. 30478041 10.1073/pnas.1812865115 PMC6294933

[msab147-B97] Neefjes J , Jongsma MLM, Paul P, Bakke O. 2011. Towards a systems understanding of MHC class I and MHC class II antigen presentation. Nat Rev Immunol. 11(12):823–836. 22076556 10.1038/nri3084

[msab147-B98] Norman PJ , Hollenbach JA, Nemat-Gorgani N, Marin WM, Norberg SJ, Ashouri E, Jayaraman J, Wroblewski EE, Trowsdale J, Rajalingam R, et al 2016. Defining KIR and HLA Class I genotypes at highest resolution via high-throughput sequencing. Am J Hum Genet. 99(2):375–391. 27486779 10.1016/j.ajhg.2016.06.023 PMC4974113

[msab147-B99] Norman PJ , Norberg SJ, Guethlein LA, Nemat-Gorgani N, Royce T, Wroblewski EE, Dunn T, Mann T, Alicata C, Hollenbach JA, et al 2017. Sequences of 95 human MHC haplotypes reveal extreme coding variation in genes other than highly polymorphic HLA class I and II. Genome Res. 27(5):813–823. 28360230 10.1101/gr.213538.116 PMC5411776

[msab147-B100] Nowak J , Mika-Witkowska R, Polak M, Zajko M, Rogatko-Koroś M, Graczyk-Pol E, Lange A. 2008. Allele and extended haplotype polymorphism of HLA-A, -C, -B, -DRB1 and -DQB1 loci in Polish population and genetic affinities to other populations. Tissue Antigens 71(3):193–205. 18179647 10.1111/j.1399-0039.2007.00991.x

[msab147-B101] Ogawa K , Okada Y. 2020. The current landscape of psoriasis genetics in 2020. J Dermatol Sci. 99(1):2–8. 32536600 10.1016/j.jdermsci.2020.05.008

[msab147-B102] Pappas DJ , Marin W, Hollenbach JA, Mack SJ. 2016. Bridging ImmunoGenomic Data Analysis Workflow Gaps (BIGDAWG): an integrated case-control analysis pipeline. Hum Immunol. 77(3):283–287. 26708359 10.1016/j.humimm.2015.12.006 PMC4828284

[msab147-B103] Parham P , Moffett A. 2013. Variable NK cell receptors and their MHC class I ligands in immunity, reproduction and human evolution. Nat Rev Immunol. 13(2):133–144. 23334245 10.1038/nri3370 PMC3956658

[msab147-B104] Park YH , Remmers EF, Lee W, Ombrello AK, Chung LK, Shilei Z, Stone DL, Ivanov MI, Loeven NA, Barron KS, et al 2020. Ancient familial Mediterranean fever mutations in human pyrin and resistance to *Yersinia pestis*. Nat Immunol. 21(8):857–2916. 32601469 10.1038/s41590-020-0705-6 PMC7381377

[msab147-B105] Patin E , Lopez M, Grollemund R, Verdu P, Harmant C, Quach H, Laval G, Perry GH, Barreiro LB, Froment A, et al 2017. Dispersals and genetic adaptation of Bantu-speaking populations in Africa and North America. Science 356(6337):543–546. 28473590 10.1126/science.aal1988

[msab147-B106] Patterson N , Moorjani P, Luo Y, Mallick S, Rohland N, Zhan Y, Genschoreck T, Webster T, Reich D. 2012. Ancient admixture in human history. Genetics 192(3):1065–1093. 22960212 10.1534/genetics.112.145037 PMC3522152

[msab147-B107] Patterson N , Price AL, Reich D. 2006. Population structure and eigenanalysis. PLoS Genet. 2(12):e190. 17194218 10.1371/journal.pgen.0020190 PMC1713260

[msab147-B108] Pearson K. 1900. On the criterion that a given system of deviations from the probable in the case of a correlated system of variables is such that it can be reasonably supposed to have arisen from random sampling. Philos Mag Ser. 50(302):157–175.

[msab147-B109] Peltzer A , Jäger G, Herbig A, Seitz A, Kniep C, Krause J, Nieselt K. 2016. EAGER: efficient ancient genome reconstruction. Genome Biol. 17(60):60. 27036623 10.1186/s13059-016-0918-z PMC4815194

[msab147-B110] Philip NH , Zwack EE, Brodsky IE. 2016. Activation and evasion of inflammasomes by Yersinia. Curr Top Microbiol Immunol. 397:69–90. 27460805 10.1007/978-3-319-41171-2_4

[msab147-B111] Pieters J. 2008. *Mycobacterium tuberculosis* and the macrophage: maintaining a balance. Cell Host Microbe. 3(6):399–407. 18541216 10.1016/j.chom.2008.05.006

[msab147-B112] Pingel J , Solloch UV, Hofmann JA, Lange V, Ehninger G, Schmidt AH. 2013. High-resolution HLA haplotype frequencies of stem cell donors in Germany with foreign parentage: how can they be used to improve unrelated donor searches? Hum Immunol. 74(3):330–340. 23200758 10.1016/j.humimm.2012.10.029

[msab147-B113] Pinhasi R , Fernandes D, Sirak K, Novak M, Connell S, Alpaslan-Roodenberg S, Gerritsen F, Moiseyev V, Gromov A, Raczky P, et al 2015. Optimal ancient DNA yields from the inner ear part of the human petrous bone. PLoS One 10(6):e0129102. 26086078 10.1371/journal.pone.0129102 PMC4472748

[msab147-B114] Politzer R. 1954. Plague. Geneva, Switzerland: World Health Organization monograph series.

[msab147-B115] Prugnolle F , Manica A, Charpentier M, Guégan JF, Guernier V, Balloux F. 2005. Pathogen-driven selection and worldwide HLA class I diversity. Curr Biol. 15(11):1022–1027. 15936272 10.1016/j.cub.2005.04.050

[msab147-B116] Quinlan AR. 2014. BEDTools: the Swiss-Army tool for genome feature analysis. Curr Protoc Bioinformatics. 47:11.12.1–11.12.34. 10.1002/0471250953.bi1112s47 PMC4213956 25199790

[msab147-B117] Quintana-Murci L. 2019. Human immunology through the lens of evolutionary genetics. Cell 177(1):184–199. 30901539 10.1016/j.cell.2019.02.033

[msab147-B118] R Development Core Team. 2011. R: a language and environment for statistical computing. Vienna, Austria: The R Foundation for Statistical Computing.

[msab147-B119] Radwan J , Babik W, Kaufman J, Lenz TL, Winternitz J. 2020. Advances in the evolutionary understanding of MHC polymorphism. Trends Genet. 36(4):298–311. 32044115 10.1016/j.tig.2020.01.008

[msab147-B120] Ralph PL , Coop G. 2015. Convergent evolution during local adaptation to patchy landscapes. PLoS Genet. 11(11):e1005630. 26571125 10.1371/journal.pgen.1005630 PMC4646681

[msab147-B121] Rascovan N , Sjögren K-G, Kristiansen K, Nielsen R, Willerslev E, Desnues C, Rasmussen S. 2019. Emergence and spread of basal lineages of *Yersinia pestis* during the Neolithic decline. Cell 176(1–2):295–305.10. 30528431 10.1016/j.cell.2018.11.005

[msab147-B122] Rasmussen S , Allentoft ME, Nielsen K, Orlando L, Sikora M, Sjögren K-G, Pedersen AG, Schubert M, Van Dam A, Kapel CMO, et al 2015. Early divergent strains of *Yersinia pestis* in Eurasia 5,000 years ago. Cell 163(3):571–582. 26496604 10.1016/j.cell.2015.10.009 PMC4644222

[msab147-B123] Renaud G , Slon V, Duggan AT, Kelso J. 2015. Schmutzi: estimation of contamination and endogenous mitochondrial consensus calling for ancient DNA. Genome Biol. 16(1):224. 26458810 10.1186/s13059-015-0776-0 PMC4601135

[msab147-B124] Robinson J , Halliwell JA, Hayhurst JD, Flicek P, Parham P, Marsh SGE. 2015. The IPD and IMGT/HLA database: allele variant databases. Nucleic Acids Res. 43(Database issue):D423–D431. 25414341 10.1093/nar/gku1161 PMC4383959

[msab147-B125] Robinson J , Thorvaldsdóttir H, Winckler W, Guttman M, Lander ES, Getz G, Mesirov JP. 2011. Integrative genomics viewer. Nat Biotechnol. 29(1):24–26. 21221095 10.1038/nbt.1754 PMC3346182

[msab147-B126] Rohland N , Harney E, Mallick S, Nordenfelt S, Reich D. 2015. Partial uracil-DNA-glycosylase treatment for screening of ancient DNA. Philos Trans R Soc Lond B Biol Sci. 370(1660):20130624. 25487342 10.1098/rstb.2013.0624 PMC4275898

[msab147-B127] Rohland N , Hofreiter M. 2007. Ancient DNA extraction from bones and teeth. Nat Protoc. 2(7):1756–1762. 17641642 10.1038/nprot.2007.247

[msab147-B128] Sabeti PC , Schaffner SF, Fry B, Lohmueller J, Varilly P, Shamovsky O, Palma A, Mikkelsen TS, Altshuler D, Lander ES. 2006. Positive natural selection in the human lineage. Science 312(5780):1614–1620. 16778047 10.1126/science.1124309

[msab147-B129] Sabeti PC , Varilly P, Fry B, Lohmueller J, Hostetter E, Cotsapas C, Xie X, Byrne EH, McCarroll SA, Gaudet R; International HapMap Consortium. 2007. Genome-wide detection and characterization of positive selection in human populations. Nature 449:913–918. 17943131 10.1038/nature06250 PMC2687721

[msab147-B130] Saunders PM , Vivian JP, O'Connor GM, Sullivan LC, Pymm P, Rossjohn J, Brooks AG. 2015. A bird's eye view of NK cell receptor interactions with their MHC class I ligands. Immunol Rev. 267(1):148–166. 26284476 10.1111/imr.12319

[msab147-B131] Schmidt AH , Baier D, Solloch UV, Stahr A, Cereb N, Wassmuth R, Ehninger G, Rutt C. 2009. Estimation of high-resolution HLA-A, -B, -C, -DRB1 allele and haplotype frequencies based on 8862 German stem cell donors and implications for strategic donor registry planning. Hum Immunol. 70(11):895–902. 19683023 10.1016/j.humimm.2009.08.006

[msab147-B132] Skoglund P , Storå J, Götherström A, Jakobsson M. 2013. Accurate sex identification of ancient human remains using DNA shotgun sequencing. J Archaeol Sci. 40(12):4477–4482.

[msab147-B133] Solloch UV , Lang K, Lange V, Böhme I, Schmidt AH, Sauter J. 2017. Frequencies of gene variant CCR5-Δ32 in 87 countries based on next-generation sequencing of 1.3 million individuals sampled from 3 national DKMS donor centers. Hum Immunol. 78(11–12):710–717. 28987960 10.1016/j.humimm.2017.10.001

[msab147-B134] Spyrou MA , Keller M, Tukhbatova RI, Scheib CL, Nelson EA, Andrades Valtueña A, Neumann GU, Walker D, Alterauge A, Carty N, et al 2019. Phylogeography of the second plague pandemic revealed through analysis of historical *Yersinia pestis* genomes. Nat Commun. 10(1):4470. 31578321 10.1038/s41467-019-12154-0 PMC6775055

[msab147-B135] Spyrou MA , Tukhbatova RI, Feldman M, Drath J, Kacki S, Beltrán de Heredia J, Arnold S, Sitdikov AG, Castex D, Wahl J, et al 2016. Historical *Y. pestis* genomes reveal the European Black Death as the source of ancient and modern plague pandemics. Cell Host Microbe. 19(6):874–881. 27281573 10.1016/j.chom.2016.05.012

[msab147-B136] Spyrou MA , Tukhbatova RI, Wang C-C, Valtueña AA, Lankapalli AK, Kondrashin VV, Tsybin VA, Khokhlov A, Kühnert D, Herbig A, et al 2018. Analysis of 3800-year-old *Yersinia pestis* genomes suggests Bronze Age origin for bubonic plague. Nat Commun. 9(1):2234. 29884871 10.1038/s41467-018-04550-9 PMC5993720

[msab147-B137] Stephens JC , Reich DE, Goldstein DB, Shin HD, Smith MW, Carrington M, Winkler C, Huttley GA, Allikmets R, Schriml L, et al 1998. Dating the origin of the CCR5-Delta32 AIDS-resistance allele by the coalescence of haplotypes. Am J Hum Genet. 62(6):1507–1515. 9585595 10.1086/301867 PMC1377146

[msab147-B138] *Sun J, Yang C, Fei W, Zhang X, Sheng Y, Zheng X, Tang H, Yang W, Yang S, Fan X, et al 2018. HLA-DQβ1 amino acid position 87 and DQB10301 are associated with Chinese Han SLE. Mol Genet Genomic Med. 6(4):541–546. 10.1002/mgg3.403 PMC6081216 29676044

[msab147-B139] Szolek A , Schubert B, Mohr C, Sturm M, Feldhahn M, Kohlbacher O. 2014. OptiType: precision HLA typing from next-generation sequencing data. Bioinformatics 30(23):3310–3316. 25143287 10.1093/bioinformatics/btu548 PMC4441069

[msab147-B140] The Genome Sequencing Consortium. 2001. Initial sequencing and analysis of the human genome. Nature 409(6822):860–921. 11237011 10.1038/35057062

[msab147-B141] Trowsdale J , Knight JC. 2013. Major histocompatibility complex genomics and human disease. Annu Rev Genomics Hum Genet. 14:301–323. 23875801 10.1146/annurev-genom-091212-153455 PMC4426292

[msab147-B142] Uhrberg M , Valiante NM, Shum BP, Shilling HG, Lienert-Weidenbach K, Corliss B, Tyan D, Lanier LL, Parham P. 1997. Human diversity in killer cell inhibitory receptor genes. Immunity 7(6):753–763. 9430221 10.1016/s1074-7613(00)80394-5

[msab147-B143] Vågene ÅJ , Herbig A, Campana MG, Robles García NM, Warinner C, Sabin S, Spyrou MA, Andrades Valtueña A, Huson D, Tuross N, et al 2018. *Salmonella enterica* genomes from victims of a major sixteenth-century epidemic in Mexico. Nat Ecol Evol. 2(3):520–528. 29335577 10.1038/s41559-017-0446-6

[msab147-B144] Vasseur E , Boniotto M, Patin E, Laval G, Quach H, Manry J, Crouau-Roy B, Quintana-Murci L. 2012. The evolutionary landscape of cytosolic microbial sensors in humans. Am J Hum Genet. 91(1):27–37. 22748209 10.1016/j.ajhg.2012.05.008 PMC3397270

[msab147-B145] Vladimer GI , Marty-Roix R, Ghosh S, Weng D, Lien E. 2013. Inflammasomes and host defenses against bacterial infections. Curr Opin Microbiol. 16(1):23–31. 23318142 10.1016/j.mib.2012.11.008 PMC3697846

[msab147-B146] Vladimer GI , Weng D, Paquette SWM, Vanaja SK, Rathinam VAK, Aune MH, Conlon JE, Burbage JJ, Proulx MK, Liu Q, et al 2012. The NLRP12 inflammasome recognizes *Yersinia pestis*. Immunity 37(1):96–107. 22840842 10.1016/j.immuni.2012.07.006 PMC3753114

[msab147-B147] Voight BF , Kudaravalli S, Wen X, Pritchard JK. 2006. A map of recent positive selection in the human genome. PLoS Biol. 4(3):e72. 16494531 10.1371/journal.pbio.0040072 PMC1382018

[msab147-B148] Wagner DM , Klunk J, Harbeck M, Devault A, Waglechner N, Sahl JW, Enk J, Birdsell DN, Kuch M, Lumibao C, et al 2014. *Yersinia pestis* and the plague of Justinian 541-543 AD: a genomic analysis. Lancet Infect Dis. 14(4):319–326. 24480148 10.1016/S1473-3099(13)70323-2

[msab147-B149] Wang ET , Kodama G, Baldi P, Moyzis RK. 2006. Global landscape of recent inferred Darwinian selection for *Homo sapiens*. Proc Natl Acad Sci U S A. 103:135–140. 16371466 10.1073/pnas.0509691102 PMC1317879

[msab147-B150] Weese D , Holtgrewe M, Reinert K. 2012. RazerS 3: faster, fully sensitive read mapping. Bioinformatics 28:2592–2599. 22923295 10.1093/bioinformatics/bts505

[msab147-B151] Weir BS , Cockerham CC. 1984. Estimating F-statistics for the analysis of population structure. Evolution 38(6):1358–1370. 10.1111/j.1558-5646.1984.tb05657.x 28563791

[msab147-B153] Wilson MJ , Torkar M, Haude A, Milne S, Jones T, Sheer D, Beck S, Trowsdale J. 2000. Plasticity in the organization and sequences of human KIR/ILT gene families. Proc Natl Acad Sci U S A. 97(9):4778–4783. 10781084 10.1073/pnas.080588597 PMC18309

[msab147-B154] Zaki MH , Man SM, Vogel P, Lamkanfi M, Kanneganti TD. 2014. Salmonella exploits NLRP12-dependent innate immune signaling to suppress host defenses during infection. Proc Natl Acad Sci U S A. 111(1):385–390. 24347638 10.1073/pnas.1317643111 PMC3890849

