# Supplementary material for: Analysis of Genomic DNA from Medieval Plague Victims Suggests Long-Term Effect of Yersinia pestis on Human Immunity Genes
Source: Mol Biol Evol. 2021 May 18;38(10):4059–76. doi: 10.1093/molbev/msab147 (PMC8476174; doi:10.1093/molbev/msab147)
Supplement: msab147_Supplementary_Data [file msab147_supplementary_data.zip › Supplementary Note 1.pdf]

Supplementary Note 1

Three early modern mass graves indicative of the plague outbreaks were analyzed yielding a total of 101 deposited individuals, of which only 24 had reached adult age. The mass graves were dated using C14 technique. Grave 549 dates between 1414-1638 cal. AD, tomb 559 covers the period between 1450-1632 cal. AD, and grave 706 dates between 1473-1634 cal. AD. The individuals were buried close to each other and there is hardly any sediment between the individual layers. The age and sex determination of the individuals were assessed by macroscopic methods. Diseases of the periodontium and the teeth, nonspecific stress markers and deficiencies, degenerative transformations, inflammatory bone changes and trauma were recorded. Furthermore, the body height of the adult individuals was reconstructed and the growth course of the sub adult individuals was analyzed.

The 101 individuals are distributed among the different age groups as follows: 2 new born (aged 0 – 1 years old (yo)), 57 children (2 – 12 yo), 19 juveniles (13 – 20 yo), and 23 adults (21 yo and older). The sex could only be determined with a degree of certainty from juveniles onwards. These remaining 42 individuals are composed of 15 women, 22 men and five undetermined individuals.

High caries frequencies may indicate that this section of Ellwangen´s population consumed carbohydrate-rich foods. Other pathological changes of the teeth and jaws such as periodontal disease, dental calculus, abrasion or intravital tooth loss are present and argue for poor oral hygiene and abrasive food.

Unspecific stress markers and deficiency symptoms were detected in subadult male and female individuals: Cribra orbitalia (46.5% of all affected individuals), enamel hypoplasia (40.7%), periostitis (15.8%) and rickets (9%). This suggests that female and male individuals were similarly exposed to diet and weaning stress during their childhood.

Degenerative changes in the joints and spine of adult individuals from Ellwangen show gender-related differences. On average, men show a higher burden of arthrosis than women, which may be related to an increased stress and a possible gender-specific division of labor. Inflammatory and infectious lesions appear rarely on the bone.

No signs of warlike violence such as blows and stab wounds could be recorded. Only two individuals show bone fractures. The few injuries suggest that they are likely to be isolated cases caused by accidents. The population of Ellwangen therefore seems to have been of a rather peaceful nature in the early modern period.

The high proportion of nonspecific stress markers in the skeletal remains of Ellwangen (Jagst) suggest that this population, albeit only seasonally, was struggling with nutrient deficiencies. Despite the numerous signs of shortages, these seem to have had no effect on the body height growth of the children from Ellwangen. Examination of the growth curves using the long bone measurements revealed no growth interruptions, nor are the long bone measures smaller than comparable samples of early modern children. Reconstruction of the body heights of the adult individuals present in the mass grave gave values of an average of 163.1 cm for women and 169.9 cm for men.
