# Supplementary material for: Analysis of Genomic DNA from Medieval Plague Victims Suggests Long-Term Effect of Yersinia pestis on Human Immunity Genes
Source: Mol Biol Evol. 2021 May 18;38(10):4059–76. doi: 10.1093/molbev/msab147 (PMC8476174; doi:10.1093/molbev/msab147)
Supplement: msab147_Supplementary_Data [file msab147_supplementary_data.zip › Supplementary_Figures_and_Tables.pdf]

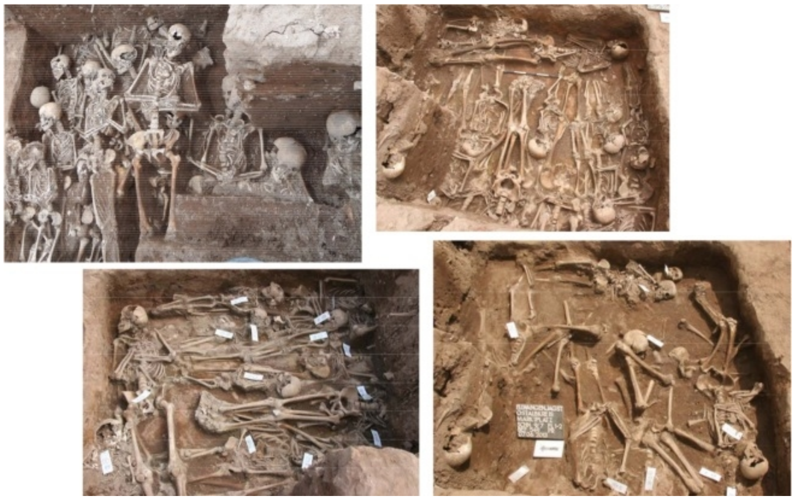

A

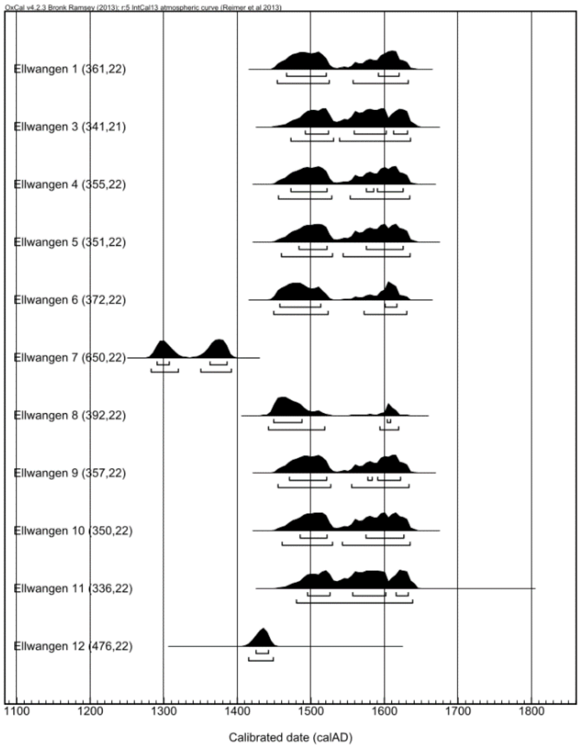
OxCal v4.2.3 Bronk Ramsey (2013); r:5 IntCal13 atmospheric curve (Reimer et al 2013)
Ellwangen 1 (361,22)
Ellwangen 3 (341,21)
Ellwangen 4 (355,22)
Ellwangen 5 (351,22)
Ellwangen 6 (372,22)
Ellwangen 7 (650,22)
Ellwangen 8 (392,22)
Ellwangen 9 (357,22)
Ellwangen 10 (350,22)
Ellwangen 11 (336,22)
Ellwangen 12 (476,22)
1100
1200
1300
1400
1500
1600
1700
1800
Calibrated date (calAD)

B

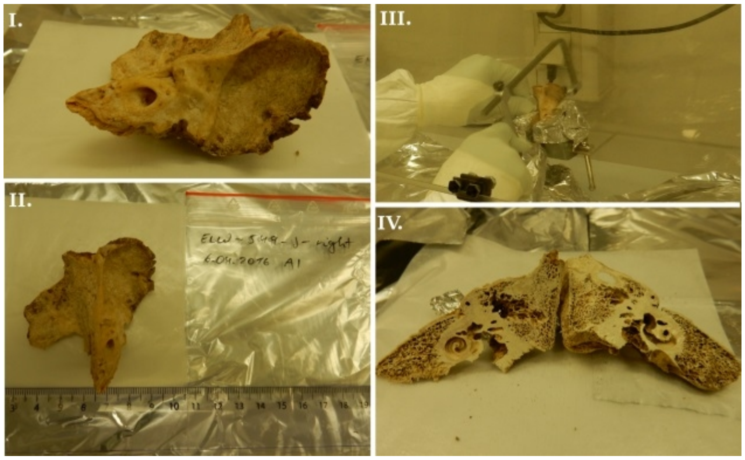
I.
II.
III.
IV.

C

Supplementary Figure 1. Archeological specimens.

A: Mass grave 549 shown in the picture, was discovered during an excavation led by the ministry for the preservation of monuments (*Landesamt für Denkmalpflege*) on the market place in Ellwangen, Baden-Wuerttemberg, in 2014/2015. Apart from mass grave 549, mass graves 559 and 706 were also excavated (pictures not shown).

B: Radiocarbon dating of bone material from 11 selected Ellwangen individuals. The black plots represent probability density functions for the calibrated C14 dates. Except one sample (Ellwangen 7) that falls into the range of the fourteenth century AD the majority of the samples fall into the sixteenth century. Ellwangen 7 was excluded from the analyses.

C: I and **II**. A petrous portion of the temporal bone of a plague victim from the mass grave 549. **III**. The petrous bone was cut longitudinally and **IV**. Powdering was done along the semi-circular canal after removing surface contamination.

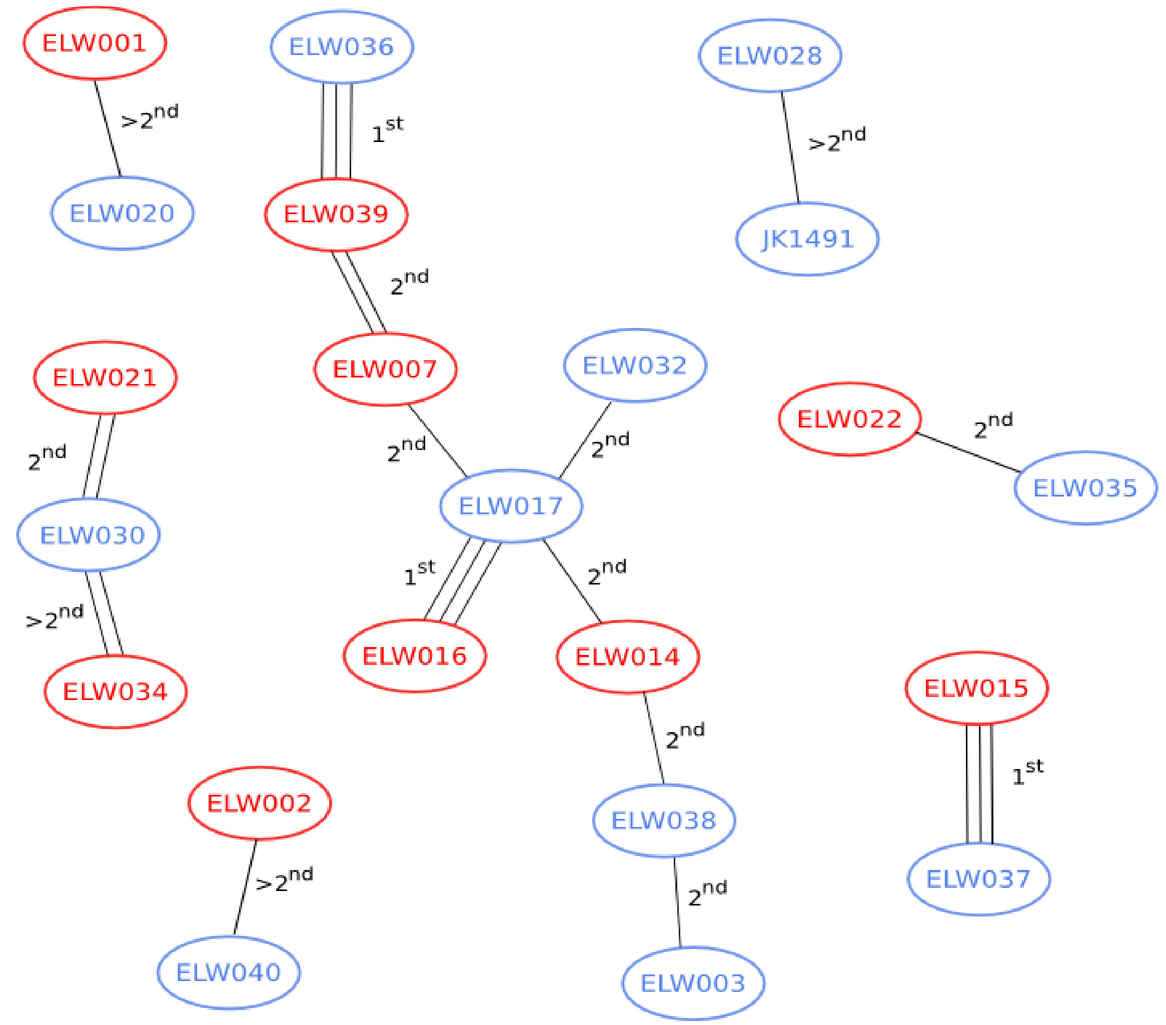

Supplementary Figure 2. Relatedness estimates among the Ellwangen plague victims.

Females are shown in red and males in blue. The number of lines joining any two individuals corresponds to the number of methods that showed relatedness; from three independent methods used. Strong support is observed for three first degree relationships: ELW015 - ELW037, ELW017 -ELW016, ELW039 - ELW036.

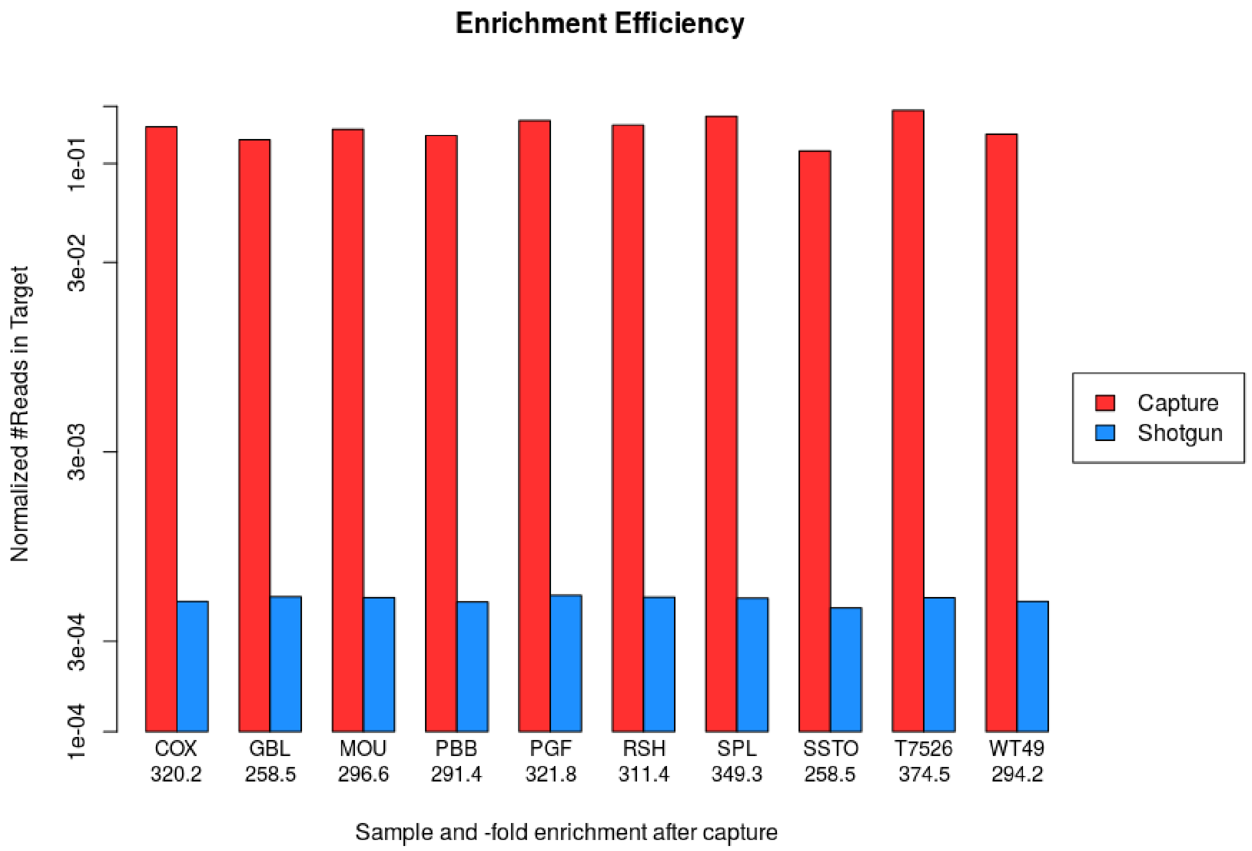

Supplementary Figure 3. Enrichment efficiency of *in solution* capture.

DNA from 10 cell lines was used, indicated by their ID names. The number of reads on target is normalized by the total number of reads and shown on a logarithmic scale for captured and whole genome sequenced libraries of the same samples. The numbers below each sample show the enrichment factor, which is the quotient of normalized reads in target after capture divided by the normalized reads in target after whole genome sequencing. The total fold enrichment was averaged for the ten samples by summing up the individual fold enrichment values and dividing by the number of samples. On total average, 308 times more reads end up in target following enrichment.

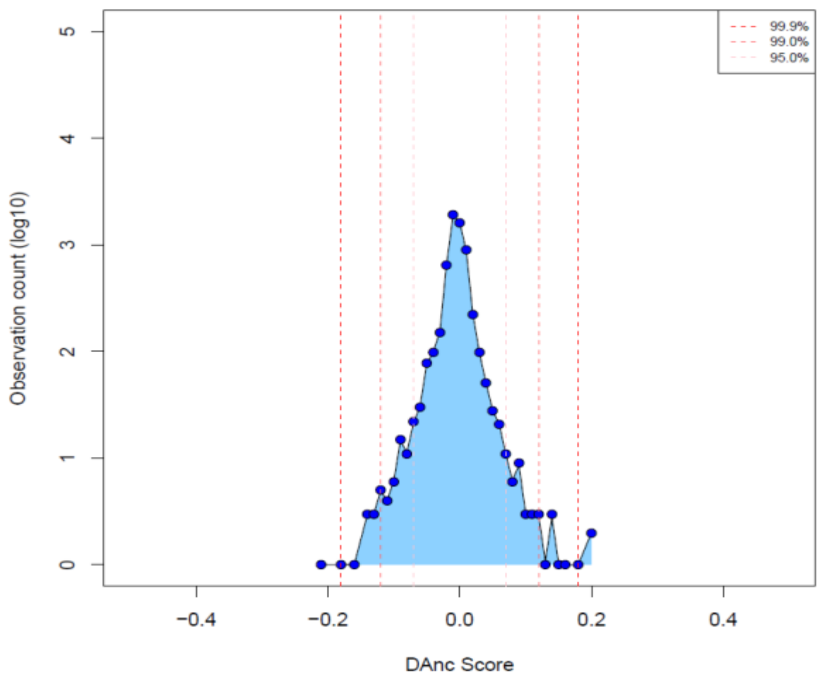

Supplementary Figure 4. Observed and simulated *DAnc* distributions. Observed *DAnc* score distribution (blue) versus simulated distribution under neutral evolution (dotted lines indicate distribution intervals). The majority of all observations fall within the 99% tail-probability indicating that observed allele frequency shifts can be explained by non-adaptive evolution.

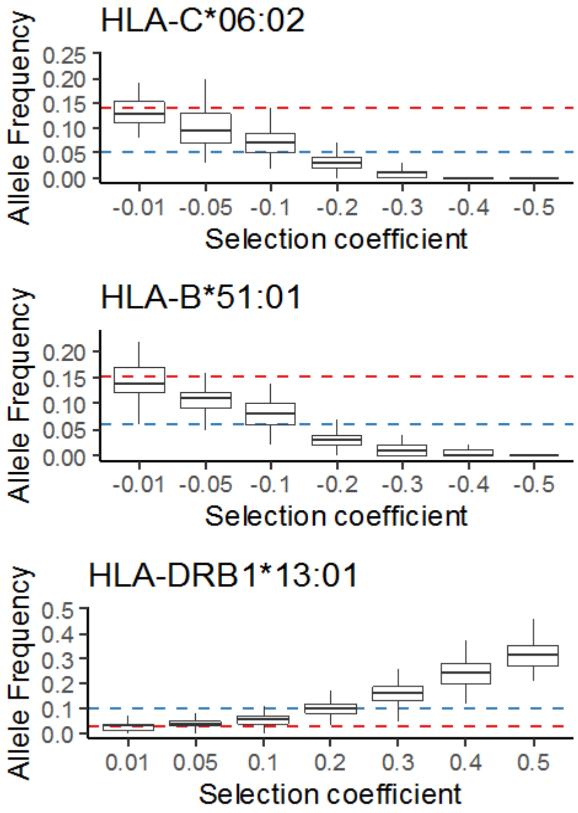

Supplementary Figure 5: Simulated selection coefficients for *HLA-C*06:02*, *-B*51:01*, and *-DRB1*13:01* alleles. Boxplots show the distribution of allele frequencies across 100 slim3 simulations following varying strengths of natural selection. Natural selection operated for 7 generations during the plague, then the allele returned to neutrality for the remainder of the simulation. The red and blue dotted lines show the observed 16th century and modern allele frequencies, respectively.

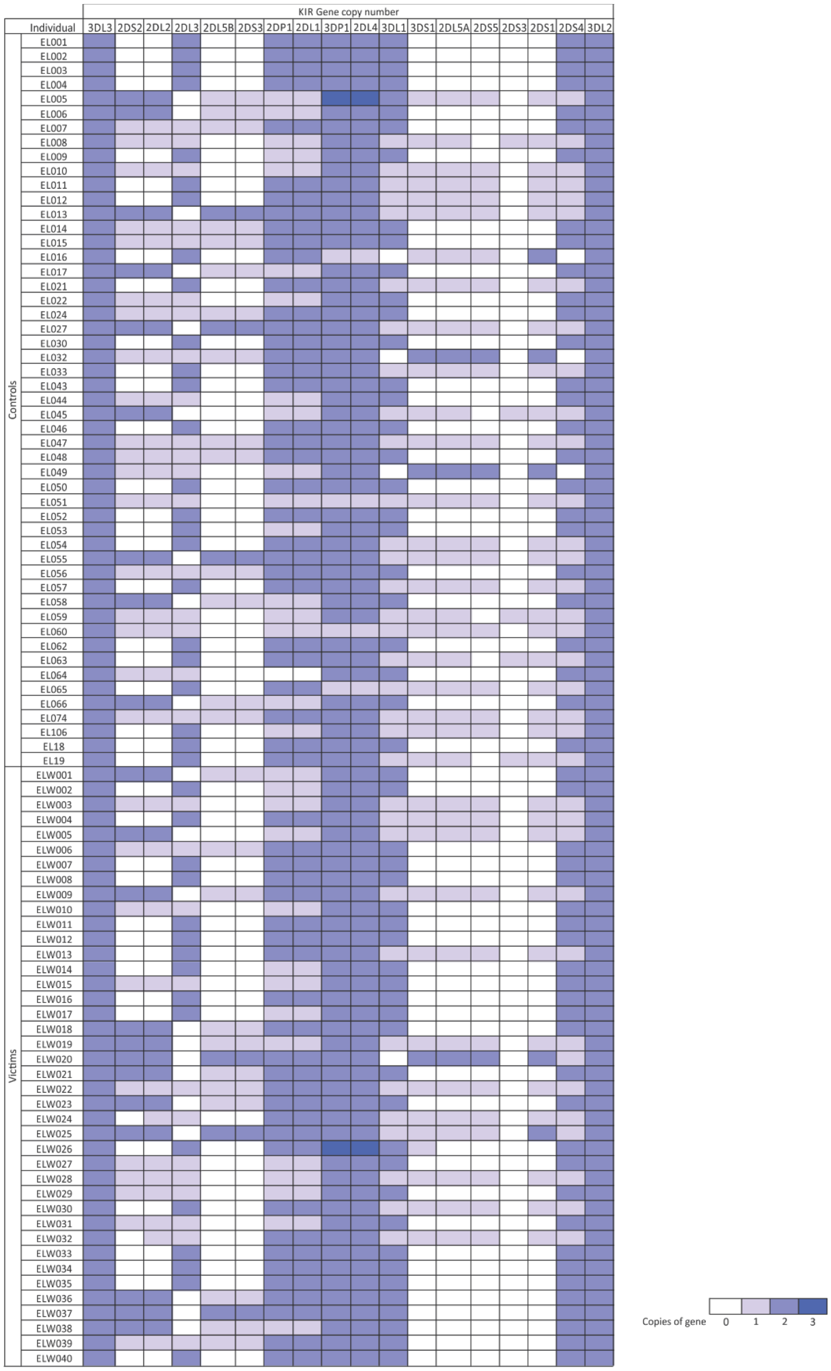

Supplementary Figure 6. KIR gene content genotypes.

Shown are the *KIR* gene content genotypes determined from the sequence reads of modern inhabitants and plague victims from Ellwangen in Germany. Colours denote the number of copies of each *KIR* gene present, and a blank square indicates the gene is absent. Nomenclature:

e.g. *3DL1* = three domains, one long cytoplasmic tail; *2DS4* = two domains, two short cytoplasmic tails. “DS” genes activate NK cells, while “DL” genes inhibit them.

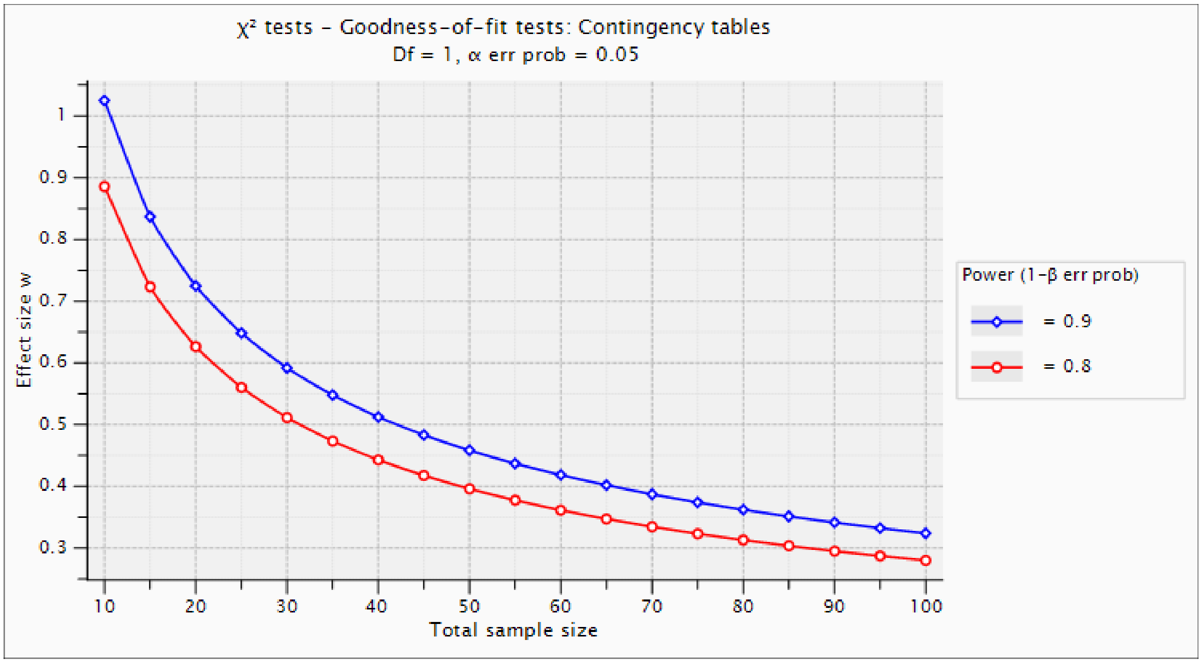

Supplementary Figure 7. Effect size analysis. Effect size as a function of sample size at a statistical power of 0.9 (blue) and 0.8 (red). With the remaining sample size of 36 ancient and 51 modern individuals, large to medium effects (*w*=0.45 - 0.4) may be observed at α=0.05 and a power of 0.8.

Supplementary Table 1. (Excel spreadsheet) Summed and assigned reads mapping to the *Y. pestis* node in MEGAN (Huson et al. 2007) are shown in brackets behind each sample positive for *Y. pestis* (summed/assigned). Blastn (version 2.7.1) was used to blast the extracted summed reads from the *Y. pestis* node against *Yersinia pestis* and *Yersinia pseudotuberculosis*. The cumulative number of gaps and mismatches from the blastn analysis is shown in brackets after the number of matching reads to *Y. pestis* and *Y. pseudotuberculosis*, respectively. Substitution frequencies for cytosine to thymine (and guanine to adenine, respectively) are shown for the 1st position from 5' end (and 3' end, respectively). The substitution frequencies underly the expected frequency distribution for sequencing libraries subjected to partial Uracil-DNA-Glycosylase ("UDG-half") treatment (Rohland, Harney, Mallick, Nordenfelt, & Reich, 2015)).

Supplementary Table 2. Genes targeted for capture.

ADPRHL2	CD180	CD86	HLA-DRA	IL22RA1	MYO5C	PSMC2	TNF
AIM2	CD19	CD8A	HLA-DRB	IL22RA2	NCAPD2	PSMC3	TNFAIP1
AIP	CD1B	CD9	HLA-E	IL23A	NFKB1	PSMC3IP	TNFAIP2
APBA2	CD1D	CD93	HLA-F	IL23R	NFKB2	PSMC4	TNFAIP3
APMAP	CD1E	CD96	HLA-G	IL24	NFKBIA	PSMC5	TNFAIP6
APOH	CD2	CD97	HLA-H	IL25	NFKBIB	PSMC6	TNFAIP8
ARID3B	CD200	CDKN2B	HLA-J	IL26	NFKBID	PSMD1	TNFAIP8L1
ARL5A	CD200R1	CFH	HLA-K	IL27RA	NFKBIE	PSMD10	TNFAIP8L2
ARPC1A	CD200R1L	CFHR5	HLA-L	IL28RA	NFKBIL1	PSMD10P	TNFAIP8L3
ARPC1B	CD207	CFI	HLA-V	IL2RA	NFKBIL2	PSMD11	TNFRSF10A
ATP5J2	CD209	CLEC10A	HSPD1	IL2RB	NFKBIZ	PSMD12	TNFRSF10B
ATP5J2-PTCD1	CD22	CLEC12A	IFI27	IL2RG	NLRB1	PSMD13	TNFRSF10C
BMP3	CD226	CLEC12B	IFIH1	IL3	NLRC3	PSMD14	TNFRSF11A
BPI	CD244	CLEC1B	IFIT1	IL31	NLRC4	PSMD2	TNFRSF11B
BUD31	CD247	CLEC2A	IFIT2	IL31RA	NLRC5	PSMD3	TNFRSF12A
C2	CD248	CLEC4A	IFIT3	IL33	NLRP1	PSMD4	TNFRSF13B
C3	CD27	CLEC4C	IFIT5	IL34	NLRP10	PSMD5	TNFRSF13C
CARD15	CD274	CLEC4D	IFNA8	IL4	NLRP11	PSMD6	TNFRSF14
CASP1	CD276	CLEC4E	IFNAR1	IL4I1	NLRP12	PSMD7	TNFRSF17
CCL1	CD28	CLEC5A	IFNAR2	IL4R	NLRP13	PSMD9	TNFRSF18
CCL11	CD2AP	CLEC6A	IFNB1	IL5	NLRP14	PSME1	TNFRSF19
CCL14	CD2BP2	CLEC7A	IFNE	IL5RA	NLRP2	PSME2	TNFRSF1B
CCL15	CD300A	CLEC9A	IFNG	IL6	NLRP3	PSME3	TNFRSF21
CCL16	CD300C	COL3A	IFNGR1	IL6R	NLRP4	PSME4	TNFRSF25
CCL17	CD300E	COL8A2	IFNGR2	IL6ST	NLRP5	PSMF1	TNFRSF4
CCL18	CD302	COQ4	IFNK	IL7	NLRP6	PSMG1	TNFRSF6B
CCL19	CD320	CPSF3L	IL10	IL7R	NLRP7	PSMG4	TNFRSF8
CCL2	CD33	CPSF4	IL10RA	IL8	NLRP8	PTCD1	TNFRSF9
CCL20	CD34	CR1	IL10RB	IL8RA	NLRP9	PTX3	TNFSF10
CCL21	CD36	CTLA4	IL11RA	IL8RB	NLRX1	PYCARD	TNFSF13
CCL22	CD37	CUX2	IL12A	IL9	NOD1	RIPK1	TNFSF13B
CCL23	CD38	CXCR3	IL12B	IRAK1	NOD2	RIPK2	TNFSF14
CCL24	CD3D	CXCR4	IL12RB1	IRAK1BP1	ODF2L	RSPO4	TNFSF15
CCL25	CD3E	CXCR5	IL12RB2	IRAK2	OXSM	SARM1	TNFSF18
CCL27	CD3G	CXCR6	IL13	IRAK3	PARD3B	SCNN1D	TNFSF4
CCL28	CD4	CXCR7	IL13RA1	IRAK4	PARK2/PACRG	SENP8	TNFSF8
CCL5	CD40	DC-SIGN/CD209	IL13RA2	KIR2DL1	PCMTD1	SLC11A1	TNRFSF1A
CCL7	CD44	DCTN1	IL15	KIR2DL4	PDAP1	SLC12A1	TOLLIP
CCL8	CD46	DDX58	IL15RA	KIR2DS4	PGL1	SLC24A5	TRAF1
CCNL2	CD47	DEFB1	IL16	KIR3DL2	PI3K	SLC27A4	TRAF2
CCR1	CD48	DUT	IL17A	KIR3DL3	PPARD	STK17B	TRAF3
CCR10	CD5	DVL1	IL17B	L1CAM	PPP2R5C	SVIL	TRAF3IP1
CCR3	CD52	EDNRA	IL17C	LAMA2	PSMA1	TAP1	TRAF3IP2
CCR4	CD53	EEF2K	IL17D	LBP	PSMA2	TAP2	TRAF3IP3
CCR5	CD55	ERAP2	IL17F	LILRA5	PSMA3	TEKT2	TRAF4
CCR6	CD59	FAM200A	IL17RA	LILRA6	PSMA4	TGFB1	TRAF5
CCR7	CD5L	FCN2	IL17RB	LILRB4	PSMA5	TICAM1	TRAF6
CCR8	CD6	GOLGA2	IL18	LILRB5	PSMA6	TICAM2	TRAF7
CCR9	CD63	GS1-259H13_2	IL18R1	LINS	PSMA7	TIRAP	TRAFD1
CCRK	CD68	HIF1A	IL19	LRP3	PSMA8	TLR1	TRAPPC3
CCRL1	CD69	HLA-A	IL1A	LTA	PSMB1	TLR10	TRUB2
CCRL2	CD7	HLA-B	IL1B	LTA4H	PSMB10	TLR2	UNC93B1
CCRN4L	CD70	HLA-C	IL1R1	MAVS	PSMB2	TLR3	URM1
CD109	CD72	HLA-DMA	IL1R2	MBL2	PSMB3	TLR4	VDR
CD14	CD74	HLA-DMB	IL2	MCP1	PSMB4	TLR5	VWA3A
CD151	CD79B	HLA-DOA	IL20	MD2	PSMB5	TLR6	WDR88
CD160	CD80	HLA-DOB	IL20RA	MIC-A	PSMB6	TLR7	ZBP1
CD163	CD81	HLA-DPA	IL20RB	MIC-B	PSMB7	TLR8	ZKSCAN5
CD163L1	CD82	HLA-DPB	IL21	MRC2	PSMB8	TLR9	ZNF655
CD164	CD83	HLA-DQA	IL21R	MXRA8	PSMB9	TMEM173	ZNF789
CD164L2	CD84	HLA-DQB	IL22	MYD88	PSMC1	TMEM30B	ZSCAN25

Supplementary Table 3. Proportion of *DAnc* scores in tails of distribution based on neutral simulations.

Ex-pected	0.05%	0.50%	2.50%	97.50%	99.50%	99.95%
Obb-tained	0.02%	0.15%	0.83%	99.45%	99.85%	99.97%

Supplementary Table 4. SNPs associated with selection through *Y. pestis* (Al Nabhani, Dietrich, Hugot, & Barreau; Laayouni et al., 2014). No significant changes between allele frequencies could be determined, respectively.

GENE	SNP	CHR	POS	16th century Ellwangen (1st allele)	Modern Ellwangen (1st allele)	16th century Ellwangen (2nd allele)	Modern Ellwangen (2nd allele)
TLR4	rs4986791	9	120475602	C:0.935897	C:0.909091	T:0.0641026	T:0.0909091
TLR4	rs4986790	9	120475302	A:0.934211	A:0.907407	G:0.0657895	G:0.0925926

Supplementary Data 1. (Excel spreadsheet) Sequencing and kinship metrics.

A: Sequencing metrics

At the left shows the total number of unique sequence reads per individual that map to the human genome following duplicate removal (Rmdup). The center columns indicate values obtained from sequencing the DNA libraries; **Raw Reads**: No. of unprocessed reads after sequencing. **Mapped Reads after rmdup**: No. of unique reads after duplicate removal, mapping to hg19. **Endogenous DNA %**: percentage of raw reads mapping to hg19. **Mean Cov**: Average coverage of hg19. **MT/NUC**: Mitochondrial to nuclear sequence ratio. **DMG 1st base 5'**: frequency of

C→T substitutions at the 1st position from 5' end (*mapDamage2.0*). **Mt Cont**: contamination estimation based on mtDNA using *Schmutzi*. **X Cont**: contamination estimation in males based on X-chromosome using *ANGSD*. The columns at the right indicate the values obtained from sequencing the libraries following enrichment with the immune capture probes; **CF** (Cluster Factor): The quotient of all mapped reads divided by the number of mapped reads after duplicate removal – the higher this number is, the more duplicates, i.e. the more identical DNA copies are present and the lower the complexity. **Reads on Target**: No. of reads mapping to the targeted regions. **Per Region Avg Readcov**: average coverage of target regions. **% target region covered>=1 (5,12,20) x after rmdup**: percentage of the targeted regions covered by unique reads at least once or 5, 12, 20 times, respectively. Negative controls: E* and EX* -extraction blanks, LIB and LB –Library blanks.

B: Kinship

From left to right: **SNPs called**: No. of SNPs called from the 1233013 (1240K) data set. **Kinship READ (lcMLkin, f3)**: relatedness estimations using the programs *READ* (*lcMLkin, f3 outgroup statistics*). **pi_Hat**: coefficient of relatedness. **f3**: measure of shared genetic drift. **NA**: not available/missing data.

Supplementary Data 2. (Excel spreadsheet) Sequence coverage of targeted genetic regions

Number of total mapped reads, cumulative length of the targeted regions and the local average sequence coverage are shown for every targeted gene.

Supplementary Data 3. Obtained *DAnc* scores for the analyzed variants according to (Key et al. 2016).

Supplementary Data 4. *DAnc* values compared to empirical Fst values.

Supplementary Data 5. (Excel spreadsheet) Test for pairwise proportion differences of HLA allele frequencies

A. Shown are frequencies of *HLA-A*, *HLA-B*, *HLA-C* and *HLA-DRB1* alleles (as well as the *CCR5- Δ32* frequency) obtained from the Ellwangen plague victims and the modern reference population subjected to a significance test for pairwise proportions. Highlighted in red are significant differences obtained prior to multiple testing correction (Benjamini-Hochberg).

B. Shown are frequencies of *HLA-A, HLA-B, HLA-C* and *HLA-DRB1* alleles obtained from the Ellwangen plague victims compared to a reference panel of 8,862 German bone marrow donor registry volunteers (Schmidt et al. 2009) subjected to a significance test for pairwise proportions. Highlighted in red are significant differences obtained before and after multiple testing correction (Benjamini-Hochberg).

Supplementary Data 6. (Excel spreadsheet) Individual genotypes and allele frequencies for *CCR5, HLA* and *KIR*.

A. *CCR5- Δ32*: genotype of the *CCR5* chemokine receptor. (wt) *CCR5*-wildtype, (Δ32) *CCR5-Δ32*, (het) heterozygous. NA: not available/missing data.

B. *HLA* genotypes. Shown are the *HLA* alleles and haplotypes present for each individual from both populations, as well as the total allele frequencies and haplotype counts.

C. *KIR* genotypes. Shown are the alleles present for *KIR3DL1/S1* as well as the flanking genes of the *KIR* locus. Blank box indicates the *KIR* gene is not present (Uhrberg et al. 1997). Colors indicate shared haplotypes. *KIR3DS1* encodes an activating receptor and occurs allelic to *KIR3DL1*; some individuals (e.g. EL5 and ELW26) have more than two alleles, due to gene

duplication (Norman et al. 2009).

Supplementary Data 7. (Excel spreadsheet) HLA site analysis.

A. Shown are frequencies of the alternative amino acids at polymorphic sites through each of the HLA molecules, in the ancient and modern Ellwangen individuals. Bold red colour indicates those differences that are significant at p<0.05. binned – low frequency (<5%) variants combined.

B. Shown are the three sites identified as significant, and the amino acid residues present in the alleles detected in the ancient (left) and modern (right) individuals.

Supplementary Data 8. Probe Sequences used for the in-solution immunity capture.

Supplementary Data 9. Neutral Evolution Model: model of the European demographic history (Gravel et al. 2011). The model was simulated using the software *slim2* (Haller et al. 2017) in order estimate the expected distribution of the obtained *DAnc* scores under neutral evolution.

Literature

Uhrberg, M. *et al.* Human diversity in killer cell inhibitory receptor genes. *Immunity* **7**, 753-763 (1997).

Norman, P. J. *et al.* Meiotic recombination generates rich diversity in NK cell receptor genes. *Genome Res.* **19**, 757-769 (2009).

Schmidt, A. H. *et al.* Estimation of high-resolution HLA-A, -B, -C, -DRB1 allele and haplotype frequencies based on 8862 German stem cell donors and implications for strategic donor registry planning. *Hum. Immunol.* **70**, 895-902 (2009).

Gravel, S. *et al.* Demographic history and rare allele sharing among human populations. *Proc. Natl. Acad. Sci. U.S.A.* **108**, 11983-11988 (2011).

Key, F. M., Fu, Q., Romagné, F., Lachmann, M. & Andrés, A. M. Human adaptation and population differentiation in the light of ancient genomes. *Nat Commun* **7**, 10775 (2016).

Haller, B. C. & Messer, P. W. SLiM 2: Flexible, Interactive Forward Genetic Simulations. *Mol. Biol. Evol.* **34**, 230-240 (2017).

Al Nabhani, Z., Dietrich, G., Hugot, J. P., & Barreau, F. Nod2: The intestinal gate keeper. (1553-7374 (Electronic)).

Laayouni, H., Oosting, M., Luisi, P., Ioana, M., Alonso, S., Ricaño-Ponce, I., . . . Netea, M. G. (2014). Convergent evolution in European and Rroma populations reveals pressure exerted by plague on Toll-like receptors. *Proc. Natl. Acad. Sci. U.S.A., 111*(7), 2668-2673.

Rohland, N., Harney, E., Mallick, S., Nordenfelt, S., & Reich, D. (2015). Partial uracil-DNA-glycosylase treatment for screening of ancient DNA. *Philos Trans R Soc Lond B Biol Sci, 370*(1660), 20130624. doi:10.1098/rstb.2013.0624
